# Evolutionary dynamics of codon usages for peste des petits ruminants virus

**DOI:** 10.3389/fvets.2022.968034

**Published:** 2022-08-12

**Authors:** Xin Wang, Jing Sun, Lei Lu, Fei-yang Pu, De-rong Zhang, Fu-qiang Xie

**Affiliations:** ^1^School of Stomatology, Lanzhou University, Lanzhou, China; ^2^Geriatrics Department, The Second Hospital of Lanzhou University, Lanzhou, China; ^3^Center for Biomedical Research, Northwest Minzu University, Lanzhou, China; ^4^Maxillofacial Surgery Department, The Second Hospital of Lanzhou University, Lanzhou, China

**Keywords:** peste des petits ruminants virus, information entropy, synonymous codon usage, evolutionary dynamics, PPRV

## Abstract

Peste des petits ruminants virus (PPRV) is an important agent of contagious, acute and febrile viral diseases in small ruminants, while its evolutionary dynamics related to codon usage are still lacking. Herein, we adopted information entropy, the relative synonymous codon usage values and similarity indexes and codon adaptation index to analyze the viral genetic features for 45 available whole genomes of PPRV. Some universal, lineage-specific, and gene-specific genetic features presented by synonymous codon usages of the six genes of PPRV that encode N, P, M, F, H and L proteins reflected evolutionary plasticity and independence. The high adaptation of PPRV to hosts at codon usages reflected high viral gene expression, but some synonymous codons that are rare in the hosts were selected in high frequencies in the viral genes. Another obvious genetic feature was that the synonymous codons containing CpG dinucleotides had weak tendencies to be selected in viral genes. The synonymous codon usage patterns of PPRV isolated during 2007–2008 and 2013–2014 in China displayed independent evolutionary pathway, although the overall codon usage patterns of these PPRV strains matched the universal codon usage patterns of lineage IV. According to the interplay between nucleotide and synonymous codon usages of the six genes of PPRV, the evolutionary dynamics including mutation pressure and natural selection determined the viral survival and fitness to its host.

## Introduction

Peste des petits ruminants (PPR) caused by peste des petits ruminants virus (PPRV) is a highly contagious, acute and febrile viral disease of wild and domestic small ruminants, and poses a great threat to the ruminant industry in the world (1). PPRV was classified under the genus *Morbillivirus*, family *Paramyxoviridae*, and order *Mononegavirales* (2). This is an enveloped virus containing a single negative-strand RNA genome of about 16,000 nt in length and has six transcription units encoding nucleocapsid (N), phosphoprotein (P), matrix (M), fusion (F), hemagglutinin (H) and polymerase (L) (3). H and F proteins (the two surface glycoproteins) function in the attachment and entry into the host cell. M protein is located on the inner surface of the viral membrane stabilizing the virion. N, P and L proteins are required for viral RNA polymerase activity. Because PPR produces a high mortality of up to 100% in immunologically naïve populations, it has been listed as a big threat to the development of sustainable agriculture by the Food and Agriculture Organization (FAO) and the World Organization for Animal Health (OIE) for eradication with the aim to globally eliminate PPR by 2030 (4).

The PPR outbreaks can cause high morbidity and mortality, resulting in severe economic losses in the developing countries. Hence, the analysis of epidemic tendency and evolutionary dynamics of PPRV for prevention and control remains particularly important. A Bayesian phylogenetic analysis of all PPRV lineages (I-IV) identified an ancestral PPRV and individual lineages of Nigeria for PPRV and Senegal for lineage I, Nigeria/Ghana for lineage II, Sudan for lineage III and India for lineage IV (5). In addition, some reports have put forwarded about the host range expansion and cross-species infection of PPRV (6–8). PPRV might switch hosts and spread more easily after eradication of rinderpest virus (RPV), similar to that the eradication of smallpox virus created a niche for cowpox and monkeypox viruses to cross the species barrier into humans (9). Based on PPR epidemics in large scale and rapidly spreading in China in the absence of RPV, the evolutionary dynamics of PPRV might provide potential opportunities for expanding the host range of PPRV in China to a certain extent. Previous reports on the evaluation of molecular epidemiology of PPRV, which was based on nucleotide usage patterns of small regions of partial sequences or the whole genome, were carried out and figured out some genetic variations for PPRV (5, 10, 11). Compared with nucleotide usage variations of coding sequences, the genetic codons consisting of triplets of nucleotides are generally redundant, and most of the amino acids can be coded by more than one codon. This phenomenon is referred to as “synonymous codon usage bias”. This bias acts as a key factor in modulating the efficiency and accuracy of protein production and maintaining the same amino acid sequence of the protein. The analysis of synonymous codon usage patterns reflects several evolutionary and functional factors in shaping the synonymous codon usage bias, including translational/natural selection, mutation pressure, host, genetic drift, gene expression, secondary protein structure and fine-tuning translation kinetics selection (12–21). Based on the knowledge on codon usages, optimization of synonymous codon usage can be frequently required for the efficient expression of genes in heterologous host systems (22–25). For better achievement of the general viral fitness, survival and evasion of the host immune system and evolution, the interplay between synonymous codon and amino acid usages of the virus and that of its host were thought to be the key evolutionary factors (26, 27). Therefore, knowledge of synonymous codon usage of PPRV enlightens PPRV molecular evolution and extends our insights into the regulation of viral gene expression.

## Materials and methods

### Information about full genome of PPRV

The 45 whole genome sequences of PPRV strains available were downloaded from the National Center for Biotechnology (NCBI) Genbank database, accessed on 1 September 2017 (Supplementary Table S1). Based on coding sequence annotations of the 45 PPRV strains, the six coding sequences (F, H, L, M, N and P) were extracted from the corresponding genome. According to the given coding sequences of PPRV, the following nucleotide contents were calculated for the coding sequences by MEGA 6.0 software: (1) the overall nucleotide usage patterns (T%, A%, C% and G%); and (2) the nucleotide usage patterns at the 1st, 2nd, and 3rd codon positions (T1, A1, C1, G1, T2, A2, C2, G2, T3, A3, C3, and G3%). Depending on the statistical test (One-way ANOVA), the overall nucleotide usage patterns and nucleotide usage patterns at the 1st, 2nd, and 3rd codon positions were described in each gene, respectively. To further investigate the genetic diversity of PPRV at nucleotide levels, a phylogenetic tree was constructed with all the full genome sequences by producing neighbor-joining trees with Kimura 2-parameter model of base substitution (Gamma distributed rate and between transitional and transversional substitutions) using MEGA 6.0 software (28).

### Nucleotide usage bias by information entropy method

As for the nucleotide usage bias at gene levels of the 45 PPRV strains, the normalized information entropy over the frequencies of different nucleotides in a given gene was presented by the below formula (29):


$$\begin{array}{r} Entropy=-\frac{\underset{i}{\sum }f_{i}\times \log_{2}\left(f_{i}\right)}{4} \end{array}$$

$$\begin{array}{r} f_{i}=\frac{F_{i}}{F\left(A\right)+F\left(T\right)+F\left(G\right)+F\left(C\right)} \end{array}$$

where *f*_*i*_ is the probability of the specific nucleotide (*F*_*i*_), and *F*_*i*_ is the total number of occurrences of the specific nucleotide in the target gene (*i, i* = *A, T, G* or *C*). The value of *Entropy* for nucleotide usage bias ranges from 0 to 1, representing how the dispersed contribution of these four types of nucleotides is: the higher the value is, the more uniform the nucleotide usage is; in contrast, a lower value reflects a more biased usage of nucleotides.

To further compare the nucleotide usage biases in the six coding sequences, the overall nucleotide usage biases, and the nucleotide usage biases at the 1st, 2nd, and 3rd codon positions were estimated by One-way ANOVA method in SPSS 16.0 software, respectively.

### Relative synonymous codon usage calculation

The relative synonymous codon usage (RSCU) values for the given coding sequences of the PPRV strains were calculated to quantify synonymous codon usage bias without the confounding influence of amino acid usage patterns or the length of different gene samples (30). Of note, two-thirds of PPRV strains were isolated from China and were classified into lineage IV (Supplementary Table S1). To better investigate the synonymous codon usage patterns of each gene of epidemic PPRV in China, we classified these strains into six groups (groups I–VI) for RSCU calculation and presented varied extents of synonymous codon usage bias in each group. In detail, the groups I, II and III corresponded to lineages I, II and III, respectively, and the groups IV, V and VI corresponded to lineage IV of foreign countries, China (2007–2008) and China (2013–2015), respectively. To identify synonymous codons with over-representation or under-representation, the synonymous codons with RSCU value of more than 1.6 and <0.6 were considered as over-represented or under-represented ones, respectively (21).

### Analysis for evolutionary distance between two different gene samples by RSCU data

To quantify the extent of similarity of codon usages between the two gene samples, a similarity index for *D(A,B)* was introduced into this study (31).


$$\begin{array}{r} R\left(A,B\right)=\frac{\overset{59}{\underset{i=1}{\sum }}a_{i}\times b_{i}}{\sqrt{\overset{59}{\underset{i=1}{\sum }}a_{i}\times \overset{59}{\underset{i=1}{\sum }}b_{i}}} \end{array}$$

$$\begin{array}{r} D\left(A,B\right)=\frac{1-R\left(A,B\right)}{2} \end{array}$$

where *R(A,B)* is defined as a cosine value of an included angle between *A* and *B* special vectors, meaning that the evolutionary distance between gene *A* and gene *B* at the aspect of 59 RSCU values, *a*_*i*_ is defined as the RSCU value for a specific codon in 59 synonymous codons of gene *A*, and *b*_*i*_ is termed as the RSCU value for the same codon of gene *B*. Here, the lower the *D(A,B)* value is, the higher the extent of similarity of codon usage patterns between gene *A* and gene *B*.

### Codon adaptation index for PPRV genes

The codon adaptation index (CAI) analysis of PPRV coding sequences were carried out depending on the CAIcal server (32), which was considered to be an improved CAI calculation measure, and estimated the expression level of a coding sequence in the host cell. The CAIcal webserver, freely available at http://genomes.urv.es/CAIcal, calculated the CAI for a group of viral sequences using the specific host reference set and included a complete set of tools related with codon usage adaptation. The host reference set required to calculate the CAI can be introduced in the codon usage database of the host. The synonymous codon usage patterns of host as reference, the synonymous codon usage bias with high extent represented the highest relative adaptation to the host, and coding sequences with higher CAI values should be regarded preferred over those with lower ones (32). The synonymous codon usage frequencies of *Ovis aries* (natural host of PPRV) were selected as the reference set, and the related data was obtained from the Codon Usage Database (33).

### Statistical analysis

To better estimate the role of nucleotide usage bias at different codon positions in the overall codon usage bias, the Davies-Bouldin index (*R*_*ij*_) (34) was introduced in this work. This index represented a ratio of within-group and between-group distances, and was defined as:


$$\begin{array}{r} R_{ij}=\frac{X_{i}+X_{j}}{M_{ij}} \end{array}$$

$$\begin{array}{r} M_{ij}=\sqrt{{\left(A_{i}-A_{j}\right)}^{2}+{\left(A_{i}-A_{j}\right)}^{2}} \end{array}$$

$$\begin{array}{r} X_{i}=\sqrt{\frac{\overset{T_{i}}{\underset{i=1}{\sum }}{\left(B_{i}-A_{i}\right)}^{2}}{T_{i}}} \end{array}$$

and


$$\begin{array}{r} X_{j}=\sqrt{\frac{\overset{T_{j}}{\underset{j=1}{\sum }}{\left(B_{j}-A_{j}\right)}^{2}}{T_{j}}} \end{array}$$

*X*_*i*_ is the standard deviation of the Euclidean distance between each point (*B*_*i*_) in the *i*th group and the centroid (*A*_*i*_) of the *i*th group; *X*_*j*_ means the standard deviation of the Euclidean distance between each point (*B*_*j*_) in the *j*th group and the centroid (*A*_*j*_) of the *j*th group, *T*_*i*_and *T*_*j*_ standard for the total numbers of points in the *i*th group and in the *j*th group, respectively. *M*_*ij*_ is the Euclidean distance between the centroids (*A*_*i*_ and *A*_*j*_) of the *i*th group and the *j*th group. The smaller the *R*_*ij*_ value is, the stronger the interaction between the two groups.

One-way ANOVA method was used to compare the means of two or more groups containing numerical response data using the software SPSS 16.0 for Windows, and significant difference can be identified when *p*-value was <0.05. Linear regression was used for modeling the relationship between a scalar dependent variable and one independent variable using the software GraphPad Prism 6 for Windows.

## Results

### Nucleotide usages in different genes of PPRV

**Figure 1:**
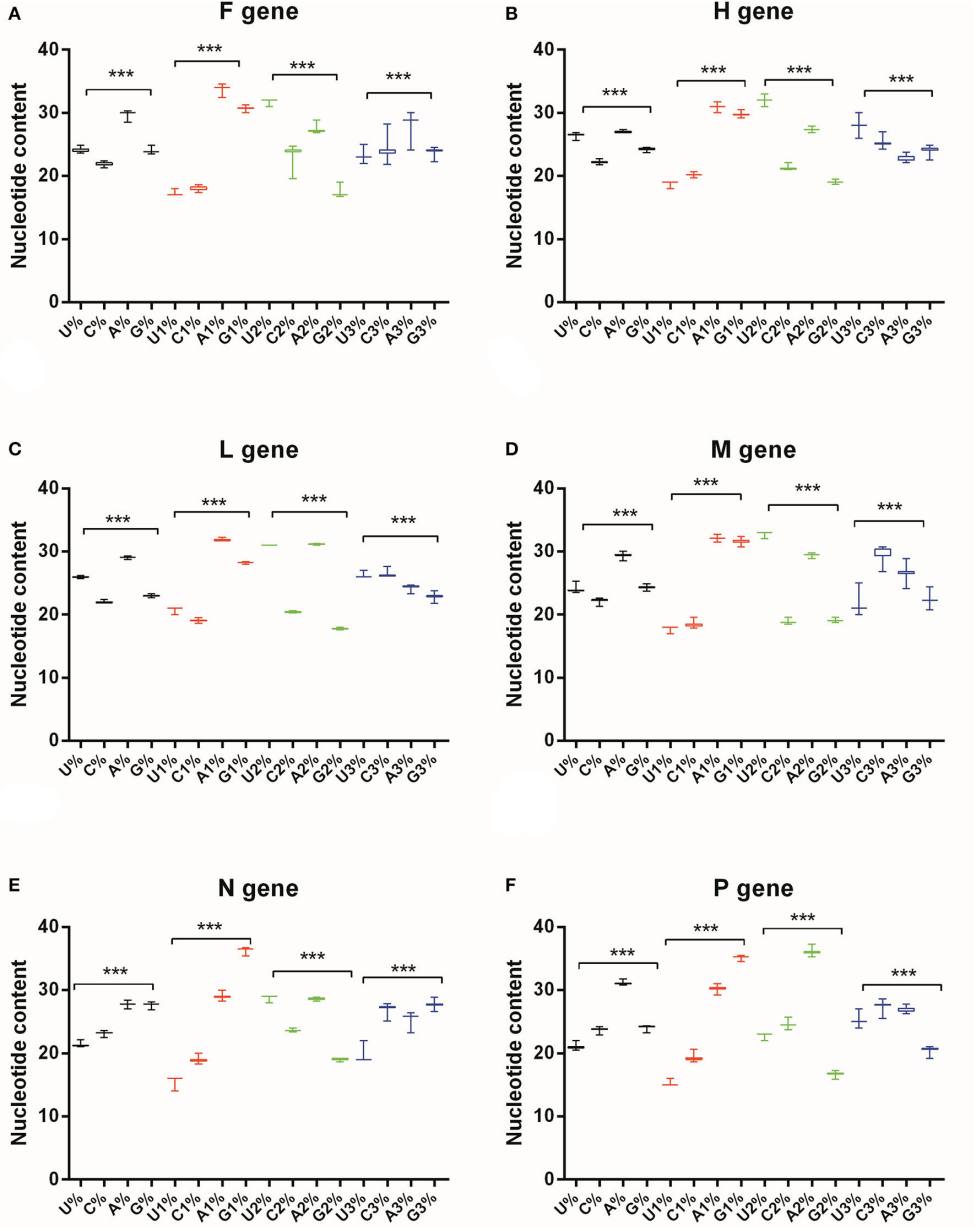
Nucleotide content of PPRV coding sequences and different codon positions. U, C, A, and G% are the overall nucleotide content of coding sequence; U1, C1, A1, and G1% are nucleotide content in the first codon position; U2, C2, A2, and G2% are nucleotide content in the second codon position; U3, C3, A3, and G3% are nucleotide content in the third codon position. The One-way ANOVA method is used for estimating the differences of nucleotide usage patterns. **(A)** F gene, **(B)** H gene, **(C)** L gene, **(D)** M gene, **(E)** N gene, **(F)** P gene. When *p*-value <0.05, it means a significant difference between the given groups. Of note, because all analyses of One-Way ANOVA in this figure produce *p*-values <0.001, they are remarked as “***”.

To quantify nucleotide composition of the six genes of PPRV, each base composition has been calculated for the 45 PPRV strains. The mean contents of A, A1, U2 and A3% were the highest in F gene (Figure 1A), the A, A1, U2, and U3% were the highest in H gene (Figure 1B), the A, A1, A2, and C3% were the highest in L gene (Figure 1C), the A, A1, U2, and C3% were the highest in M gene (Figure 1D), the A, G1, U2 and G3% were the highest in N gene (Figure 1E), and the A, G1, A2, and C3% were the highest in P gene (Figure 1F). As shown in Figure 1, there were significant differences in the overall nucleotide usages and in the first, second and third codon positions of each PPRV gene (*P* < 0.0001). Generally, the nucleotide usage patterns represented the gene-specific compositional trends rather than the similar compositional trends with the overall content of nucleotides (Supplementary Table S2).

### Nucleotide usage bias of viral gene by information entropy

**Figure 2:**
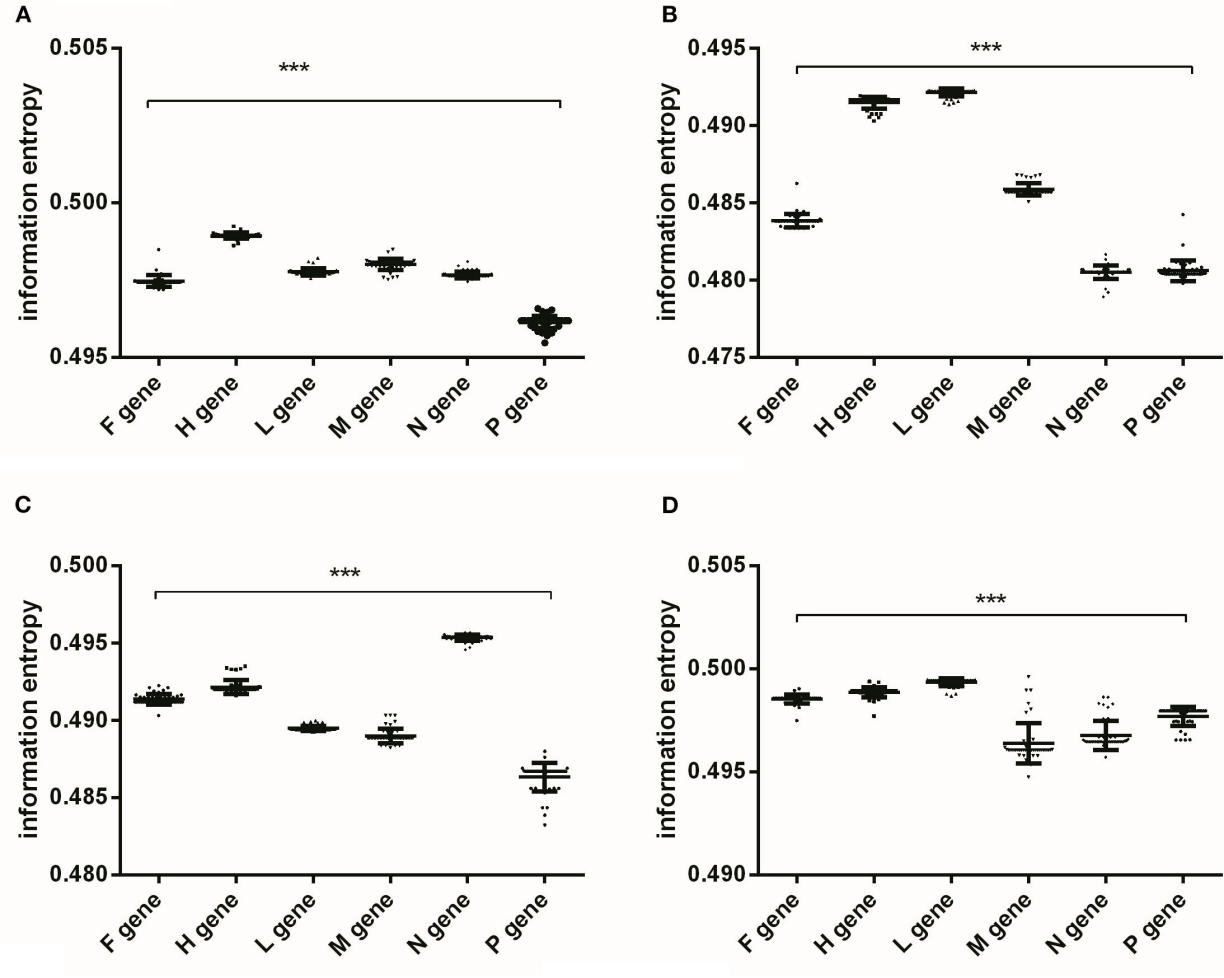
The analysis of nucleotide usage bias of PPRV coding sequences performed by information entropy. **(A)** the overall nucleotide usage bias at gene levels, and significant differences of the overall nucleotide usage biases among the six coding sequences; **(B)** the nucleotide usage bias at the first codon position, and significant differences of nucleotide usage bias at the first codon position among the six genes; **(C)** the nucleotide usage bias at the second codon position, and significant differences of nucleotide usage bias at the second codon position among the six genes; **(D)** the nucleotide usage bias at the third codon position, and significant differences of nucleotide usage bias at the third codon position among the six genes. The One-way ANOVA method is used for estimating the differences of nucleotide usage biases among the six genes, and *p*-value <0.001 remarks as “***”.

According to the variations of nucleotide content at different codon positions of PPRV genes (Figure 1), information entropy was performed to quantify nucleotide usage bias at gene levels and the bias at the three nucleotide positions of codons in viral gene. There were significant differences in the nucleotide usage biases at gene levels, the 1st, 2nd, and 3rd codon positions, respectively (Figure 2). As shown in Figure 2, the overall nucleotide usage bias in H gene was the highest (Figure 2A), the nucleotide usage bias in the 1st position in L gene was the highest (Figure 2B), the nucleotide usage bias in the 2nd position in N gene was the highest (Figure 2C), and the nucleotide usage bias in the 3rd position in L gene was the highest (Figure 2D). Nucleotide usage biases at gene levels and at different codon positions quantified by information entropy showed that the nucleotide mutations in different viral coding sequences resulted in the evolutionary dynamics. Although nucleotide usage biases at gene levels and at different codon positions represented a gene-specific characteristic (Supplementary Table S3), information entropy can comprehensively quantify the trends of nucleotide usage bias caused by four nucleotide contents and confirm the low extent of nucleotide substitution rates in the six genes of PPRV.

### Synonymous codon usage bias of PPRV coding sequences

The RSCU analysis were carried out to quantify the extent of synonymous codon usage bias in the six coding sequences of PPRV (Supplementary Tables S4–S9). All over-representative synonymous codons were not the codons associated with G/C end or A/U end, and most synonymous codons containing CpG had a weak tendency to be selected by PPRV genes (Table 1). Interestingly, compared to those synonymous codons of lineage I (Supplementary Table S8), the synonymous codons (UGU for Cys and CAU for His) were never selected by the N gene of PPRV lineages II, III and IV, indicating that the synonymous codon usage was one of the evolutionary dynamics associated with PPRV. PPRV coding sequences had a weak tendency to select synonymous codons containing CpG dinucleotides (RSCU < 1.0, Supplementary Tables S4–S8), except CGU for Arg in P gene (Supplementary Table S9). The comparisons of RSCU data for the coding sequences of groups IV, V and VI represented a stable Synonymous usage pattern in lineage IV PPRV strains of other countries, China (2007–2008) and China (2013–2015) (Supplementary Table S4–S9), suggesting that the synonymous codon usage pattern could sustain the difference between lineages of PPRV rather than that of outbreaks or countries. In addition, we adopted RSCU method for analyzing the usage pattern of the three stop codons. Despite the three canonical stop codons with the same biological function (gene translation end), the stop codon UGA was only selected by H gene and UAA was selected by P gene. Moreover, F and L genes strongly tended to select UAG as stop codon, M and N genes strongly tended to select UAA as stop codon, and UGA was never selected by F, L, M, and N genes of PPRV. These phenomena indicate that the PPRV coding sequences had evolved lineage- and gene-specific synonymous codon usage patterns.

**Table 1:** The over-/under-representative synonymous codons in the six transcriptions of PPRV genome.

	**Over-representative synonymous codons**	**Under-representative synonymous codons**
F	UCA for Ser, CCA for Pro, GGG for Gly	CCG for Pro, ACG for Thr, GCG for Ala, GGU for Gly, CGC, CGA and CGG for Arg
H	UCA for Ser, AGA and AGG for Arg	UCG for Ser, ACG for Thr, GCG for Ala, CGU, CGC and CGA for Arg
L	AGA and AGG for Arg	UCG for Ser, ACG for Thr, GCG for Ala, CGU and CGC for Arg
M	UCA for Ser, CCC for Pro, UGC for Cys and AGA for Arg	UCC and UCG for Ser, CCG for Pro, ACG for Thr, GCG for Ala, UGU for Cys, CGU and CGG for Arg
N	UCA for Ser, AGA and AGG for Arg, and GGU for Gly	UUA for Leu, AGC for Ser, ACG for Thr, GCG for Ala, CGU, CGC, CGA and CGG for Arg
P	GUC for Val and AGA for Arg	GUA for Val, UCG for Ser, ACG for Thr, GCG for Ala, CGC, CGA and CGG for Arg

### Phylogenetic analysis

**Figure 3:**
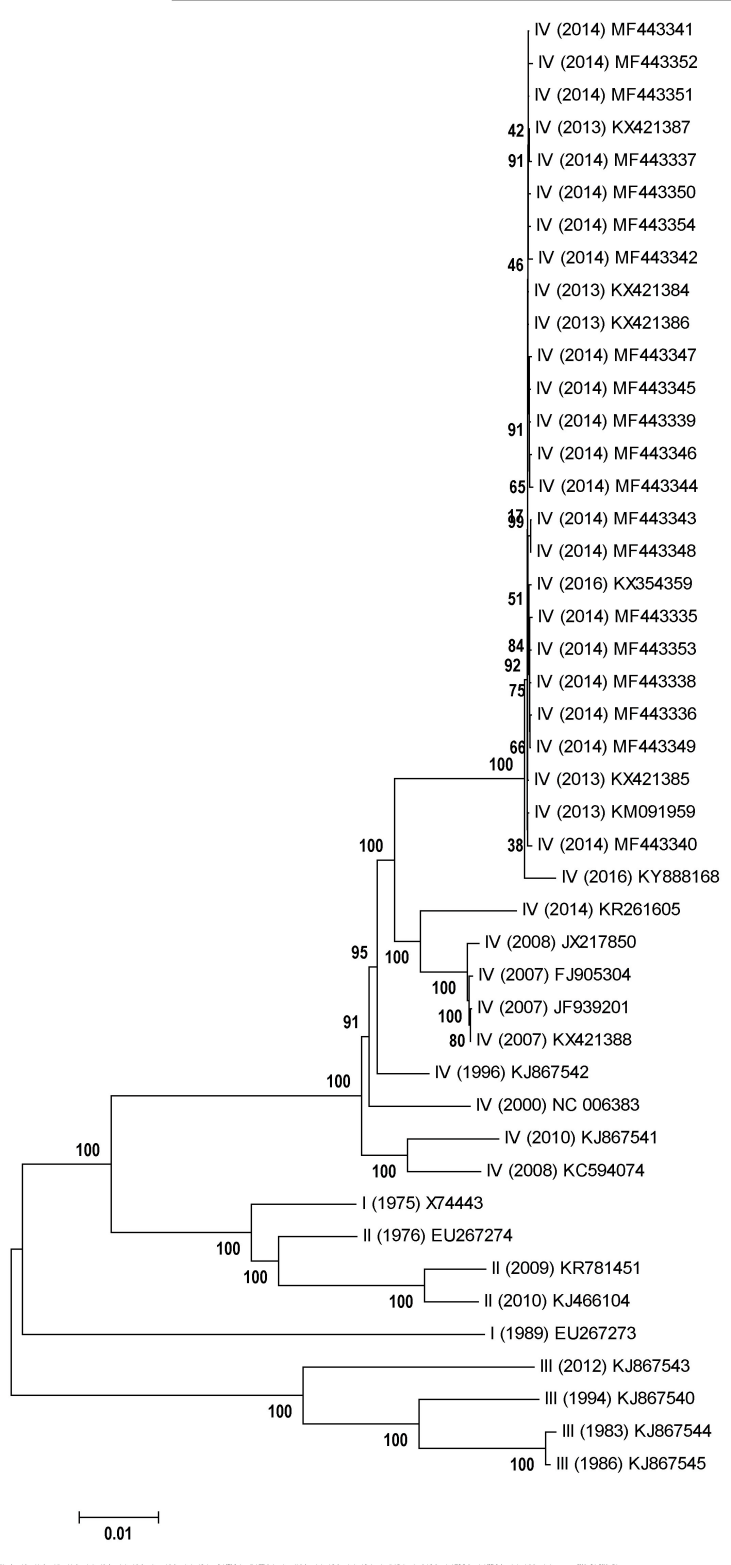
Phylogenetic relationships of PPRV genome sequences. Representation is a neighbor-joining tree of 45 PPRV genome sequences generated in this work. The phylogenetic tree is unrooted. The scale bar is given in numbers of substitutions per site. Bootstrap resampling (1,000 replications) support values are represented at the nodes.

According to phylogenetic analysis *via* neighbor-joining model, although the lineage IV was not monophyletic, this lineage was able to be separated from other three lineages (Figure 3). Furthermore, the PPRV strains isolated from China (2013–2014) could be classified into a distinct clade in lineage IV, while the strains firstly emerged in China (2007–2008) were grouped into another distinct clade in lineage IV (Figure 3). To some degree, the genetic diversity of PPRV strains isolated from China displayed the geographic trace in comparison of that of PPRV strains (KJ867542, NC_006383, KJ867541 and KC594074) isolated from non-Asia region. In addition, the PPRV strains with lineage III owned the isolated evolutionary pathway, compared with those of lineages I, II and IV. However, the two PPRV strains with lineage I displayed the obvious genetic divergence at the genome level (Figure 3).

Although the PPRV strains from China (2007–2008), China (2013–2015) and other countries generally shared a similar synonymous codon usage pattern (Supplementary Tables S4–S9), the differences of synonymous codon usage clarified the genetic divergence of the three groups [China (2007–2008), China (2013–2015) and other countries]. *D(A,B)* analysis also found highly similar extent of synonymous codon usage in viral genes of the three groups (Table 2) based on the data derived from the Supplementary Tables S4–S9. The extent of the overall codon usage similarity of lineage IV between other countries and China (2013–2015) was generally higher than that between other countries and China (2007–2008) or between China (2007–2008) and China (2013–2015) (Table 2). In addition, the extent of overall codon usage similarity of M and N genes between other countries and China (2007–2008) was less than that between China (2007–2008) and China (2013–2015) (Table 2). These results reflected that genetic divergence in synonymous codon usage of viral genes of PPRV lineage IV was complex and synonymous codon usage was able to alleviate the adverse effect by nucleotide mutation in viral genes.

**Table 2:** Evolutionary distances among strains of PPRV lineage IV isolated from China (2007–2008 and 2013–2015) and other countries.

	***D(A,B)***	***D(A,C)***	***D(B,C)***
F gene	0.0027	0.0013	0.0027
H gene	0.0020	0.0028	0.0042
L gene	0.0003	0.0005	0.0007
M gene	0.0033	0.0011	0.0020
N gene	0.0050	0.0010	0.0022
P gene	0.0024	0.0018	0.0061

A means strains isolated from other countries.

B means strains isolated from Tibet in China (2007–2008).

C means strains isolated from other provinces in China (2013–2015).

### PPRV presenting host-specific codon adaption patterns

**Figure 4:**
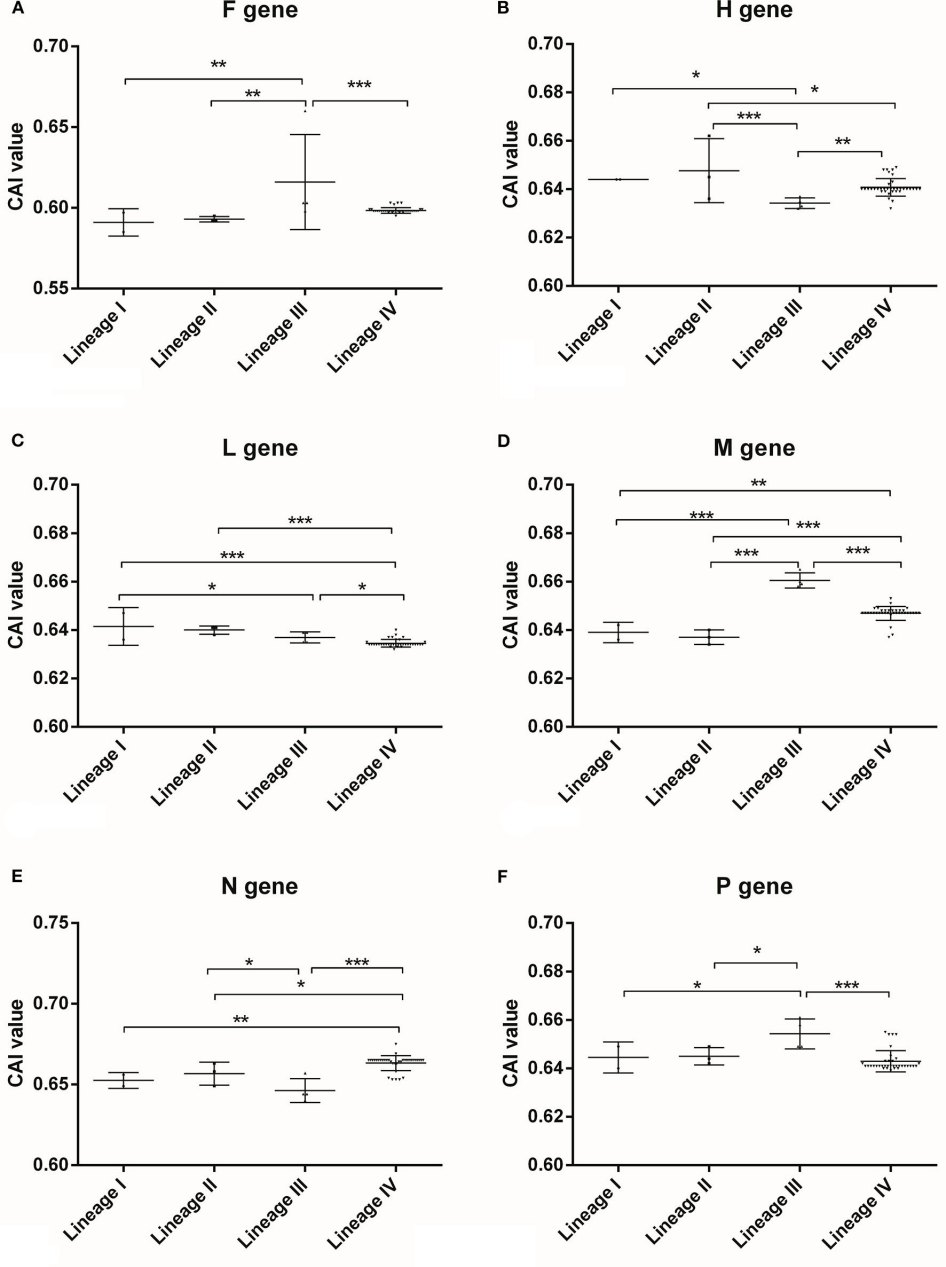
CAI analysis for PPRV coding sequences of different lineages in relation to its host performed by CAIcal server. CAI is frequently used as a measure of gene expression and to assess the adaptation of viral genes to their hosts, which indicates the influence of natural selection. The higher CAI value is, the more adaptation of synonymous codon usage of the target coding sequence is to its host. **(A)** F gene, **(B)** H gene, **(C)** L gene, **(D)** M gene, **(E)** N gene, **(F)** P gene. “*” means significant difference between the two different lineages with *p*-value <0.05 performed by One-way ANOVA method, “**” means significant difference between the two different lineages with *p*-value <0.01 performed by One-way ANOVA method, “***” means significant difference between the two different lineages with *p*-value <0.001 performed by One-way ANOVA method.

The CAI analysis were performed to estimate the correlation between the synonymous codon usage bias and the expression efficiencies of gene samples of PPRV, implying that the strong codon adaptation of the viral genes fit the host (*Ovis aries*) cellular machinery. Based on the classification of gene types, a strong significant difference was found (Supplementary Table S3), implying that the six viral genes of PPRV might have different gene expression levels in the host cells. Generally, the viral gene (N gene) had the highest expression level, while F gene owned the lowest one (Supplementary Figure S1), suggesting that PPRV had developed gene-specific codon usage patterns for adaption to the codon usage of host cellular environment. Based on the classification of lineages, significant differences were discovered among the four lineages of PPRV (Figure 4), suggesting that the six genes had developed lineage-specific codon usage patterns. To better compare the synonymous codon usage pattern between PPRV and its natural host (*Ovis aries*) based on RSCU data for *Ovis aries* (21), the six genes of PPRV generally shared a similar synonymous codon usage pattern with *Ovis aries*. However, some rare synonymous codons in *Ovis aries* were preferably selected by PPRV genes, including UCA (Ser) and AAU (Asn) in F gene, UCA (Ser) in H gene, UUG (Leu), UCA (Ser) and AAU (Asn) in L gene, CUA (Leu) and UCA (Ser) in M gene and UCA (Ser) in P gene. These synonymous codons likely mediated and regulated the relevant viral genes translation due to its low corresponding tRNA abundance in the host.

As shown in Supplementary Figure S1, the high level of adaptation of viral synonymous codon usages to that of the host implied that PPRV coding sequences can be translated at relatively high efficiencies. The Davies-Bouldin index (*Rij* value) indicated that despite the obvious effects of nucleotide usage biases at different codon positions on the overall codon usage bias of PPRV coding sequences, nucleotide usage biases at the first and second codon positions played more vital roles than that at the third codon position in the codon usage bias for the coding sequences (Table 3).

**Table 3:** Roles of nucleotide usage bias in the overall codon usage bias of PPRV genes.

	**Rij(CAI,total)**	**Rij(CAI,N1)**	**Rij(CAI,N2)**	**Rij(CAI,N3)**
F gene	0.096	0.087	0.092	0.098
H gene	0.037	0.037	0.037	0.038
L gene	0.021	0.021	0.020	0.022
M gene	0.040	0.038	0.040	0.045
N gene	0.045	0.042	0.044	0.048
P gene	0.038	0.037	0.040	0.040

The word ‘total' means the overall nucleotide usage bias; ‘N1' means nucleotide usage bias in the first codon position; ‘N2' means nucleotide usage bias in the second codon position; ‘N3' means nucleotide usage bias in the third codon position.

## Discussion

The high mutation rate of nucleotide usages in PPRV genome results in viral expansion both in geographical range and in the hosts it infects (35). It has been reported that some negative-sense single-stranded RNA viruses (such as Marburg virus and human metapneumovirus) contain all U- or A-ended codons to encode amino acids, because this synonymous codon usage pattern exhibited great association with its two nucleotides in high proportion in their genomes (36, 37). However, the nucleotide compositional patterns of the six coding sequences of PPRV were more complex than the generally analyzed AU and/or GC-rich compositions of most microorganisms. The gene-specific nucleotide usage pattern, the stable nucleotide usage patterns at the first and second codon positions and the various nucleotide usage patterns at the third codon position were obviously the genetic features of PPRV genes. This further suggests that the synonymous codon usages can be considered as the evolutionary dynamics, alleviating the effects of nucleotide usage variation in viral genes on amino acid composition of viral protein. Previous studies have supported these PPRV genetic features and that the PPRV nucleotide variation throughout the complete genome proved genome plasticity, which might explain the viral ability to emerge and adapt in new geographic regions and hosts (5, 38). Although nucleotide usage variation finally influences the biological functions of viral proteins, synonymous codon usages play a non-negligible role in viral biological functions to achieve the viral evolutionary origins and adaption to new hosts (39–42). For some microorganisms, including viruses, an AU-rich or GC-rich nucleotide composition was strongly correlated with their synonymous codon usage bias, in other words, an AU-rich genome tended to select synonymous codons with A/U ended, while a GC-rich genome strongly selected synonymous codons with G/C ended (21, 36, 43–45). If synonymous codon usage bias reflected such trends as mentioned above, the mutation pressure would play a dominant role in the codon usages. During the evolution of the negative-sense single-stranded RNA virus, mutation pressure remained the key factor that influenced the codon bias than natural selection in viral genes (46, 47). However, synonymous codon usage bias of PPRV coding sequences showed no AU or GC end, suggesting that the mutation pressure caused by nucleotide usage variation was not predominant in the PPRV evolutionary pathway. The frequencies of CpG and UpA dinucleotides played important roles in RNA virus replication and virulence, and nucleotide usage frequencies caused by dinucleotide usages meant selection pressures independent of coding capacity and profoundly influenced host-pathogen interactions (48–50). The rich CpG motif in genes can enhance immune response of the host against pathogens (51–54). Viral genes of PPRV should avoid selecting synonymous codons containing CpG dinucleotides. As for the respiratory syncytial virus, codon-optimized F gene with low level of CpG dinucleotides had higher expression of F, replicated more efficiently *in vivo*, and was more immunogenic (55). With poor CpG dinucleotides in the viral genes, PPRV can avoid stimulating strong immune responses of the host for immune escape. This suggests that apart from mutation pressure, other evolutionary dynamics related to natural selections played roles in the evolution of PPRV. Similar phenomena had been reported in influenza virus and foot-and-mouth disease virus (21, 44).

*Flaviviridae* family had a big epidemic around the world and their members had developed host- and vector-specific codon usage patterns to maintain successful replication and transmission chains within multiple hosts and vectors (56, 57). Some members of *Picornaviridae* family also demonstrated that the natural hosts played important roles in viral synonymous codon usages (13, 21). In the family *Paramyxoviridae*, codon usage patterns remained specific for each viral species and were markedly different among diverse hosts (58). CAI analysis for the six coding sequences of PPRV reflected good fitness of the virus to the host and high levels of viral gene expression in terms of codon usage pattern. Additional evidence has confirmed that usage of synonymous codons in protein coding sequences is necessarily biased and the overall codon usage pattern could match the tRNA pool of the host organism (27, 59–65). Since synonymous codon usage bias reflected tRNA abundance in host cells, and synonymous codon usage patterns of RNA virus, which was well fitted to its hosts, and might influence viral translation efficiencies (66–68). Even in the same genome, the synonymous codon usage patterns vary significantly among genes given their different expression levels, biological functions and tissue-specific patterns (69–71). Of note, some synonymous codons are preferentially selected over others at higher frequencies, resulting in synonymous codon usage bias, and is found in almost all available genomes. This biased synonymous codon usage is not neutral but involved in nucleotide usage bias (72, 73), mRNA stability (74, 75), translation accuracy, efficiency (76, 77), and protein folding formation (78).

In China, the first emergence of PPR occurred in Tibet China (2007) (79). Another outbreak of PPR occurred in wild small ruminants in Tibet China (2008) (79). PPR outbreak was not further reported until December 2013 in Xinjiang Yili (80). Although this epidemic was effectively controlled through a series of effective measures, PPR had widely and rapidly intruded into 21 provinces due to the movement of small ruminants (81). Although the two PPR epidemics in China presented genetic divergence in nucleotide usage bias and synonymous codon usage bias in viral genes, the two groups still had shared lineage-specific features in synonymous codon usage pattern. Since PPRV contains the gene for the RNA-dependent RNA polymerase NS5B in its genome and the polymerase does not have proofreading activity reading, PPRV haves a high error rate leading to genetic heterogeneity and the formation of quasispecies. Synonymous codon is regarded as a linker between nucleotide and amino acid usages, resulting in enhancing fault tolerance of PPRV proteins caused by viral quasispecies to some degree. Moreover, synonymous codon usage bias derived from the homeostasis between natural selection and mutation pressure is a universal phenomenon across the genomes of microorganisms and profoundly influences genomic evolution (36, 56).

Previously, the major bottleneck limiting in better understanding of the genetic features of PPRV was their dependence on nucleotide usage variation. Although nucleotide usage variation can be regarded as evolutionary dynamics of PPRV genome, synonymous codon usage patterns of PPRV coding sequences carry more genetic information, including viral adaptation to hosts, viral gene expression, and effects on the biological functions of viral protein.

## Data availability statement

The datasets presented in this study can be found in online repositories. The names of the repository/repositories and accession number(s) can be found in the article/Supplementary material.

## Author contributions

Conceptualization: XW, F-yP, and F-qX. Methodology: F-yP, F-qX, XW, and D-rZ. Software: XW and F-yP. Formal analyses: F-yP, D-rZ, XW, and JS. Writing-review and editing: XW, JS, and F-qX. All authors contributed to the article and approved the submitted version.

## Funding

This work was supported by Department of Science and Technology of Gansu Province Nature and Science Fund [21JR1RA141], Cuiying Scientific and Technological Innovation Program of Lanzhou University Second Hospital [CY2021-BJ-A18, CY2019-MS07], Cuiying Postgraduate Student Supervisor Culture Plan [CYDSPY202005], Innovation Foundation for Higher Education Institution of Gansu Province [2020B-022], and Gansu Province Science and Technology Fund [20JR10RA737].

## Conflict of interest

The authors declare that the research was conducted in the absence of any commercial or financial relationships that could be construed as a potential conflict of interest.

## Publisher's note

All claims expressed in this article are solely those of the authors and do not necessarily represent those of their affiliated organizations, or those of the publisher, the editors and the reviewers. Any product that may be evaluated in this article, or claim that may be made by its manufacturer, is not guaranteed or endorsed by the publisher.

## Abbreviations

PPR: Peste des petits ruminants
PPRV: Peste des petits ruminants virus
FAO: Food and Agriculture Organization
OIE: World Organization for Animal Health
NCBI: National Center for Biotechnology
RSCU: relative synonymous codon usage
CAI: codon adaptation index.
