# Supplementary material for: Evolutionary dynamics of codon usages for peste des petits ruminants virus
Source: Front Vet Sci. 2022 Aug 12;9:968034. doi: 10.3389/fvets.2022.968034 (PMC9412750; doi:10.3389/fvets.2022.968034)
Supplement: Supplementary file 1 [file Data_Sheet_1.doc]

**Evolutionary dynamics of codon usages for peste des petits ruminants virus**

Xin Wang^1†^, Jing Sun^2†^, Lei Lu^1^, Fei-yang Pu^3^, De-rong Zhang^3^, Fu-qiang Xie *^,4^

1. School of Stomatology, Lanzhou University, Lanzhou, Gansu, China

2. Geriatrics Department, The Second Hospital of Lanzhou University, Lanzhou, Gansu, China

3. Center for Biomedical Research, Northwest Minzu University, Lanzhou, Gansu, China

4. Maxillofacial Surgery Department, The Second Hospital of Lanzhou University, Lanzhou, Gansu, China

**Supplementary Figure:**


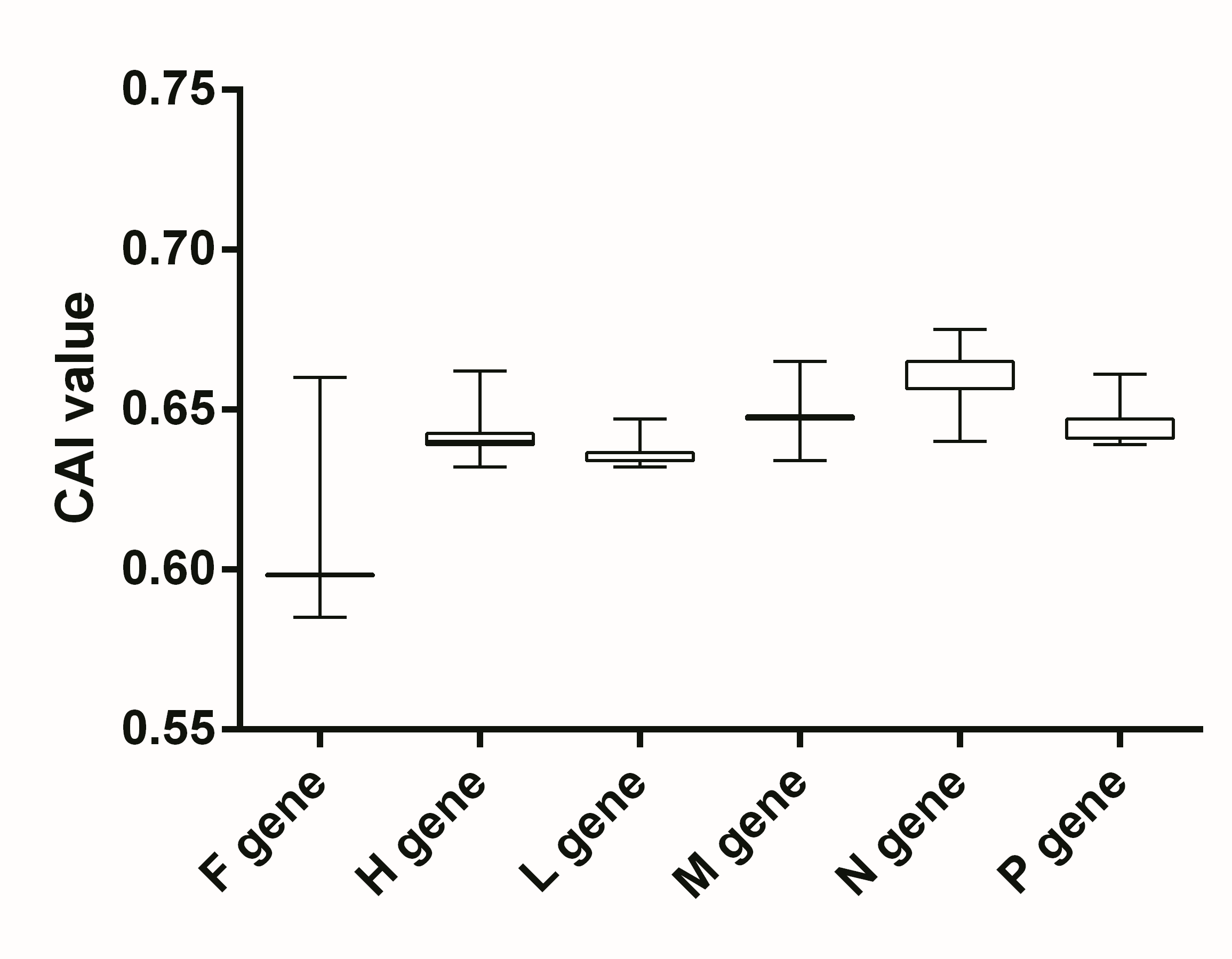


Fig. S1 CAI analysis of PPRV coding sequences in relation to its host performed by CAIcal server. CAI is frequently used as a measure of gene expression and to assess the adaptation of viral genes to thieir hosts, which indicates the influence of natural selection. The higher CAI value is, the more adaptation of synonymous codon usage of the target coding sequence is to its host.

Table S1 The information about PPRV in this study

| Strain | Country | Year | GenBank accession no. | Lineage |
| --- | --- | --- | --- | --- |
| Nigeria/75/1 | Nigeria | 1975 | X74443 | I |
| Sungri 1996 MSD | India/Sungri | 1996 | KJ867542 | IV |
| China/Tib/07 | China/Tibet | 2007 | JF939201 | IV |
| China/Tibet/30/2007 | China/Tibet | 2007 | FJ905304 | IV |
| China/33/2007 | China/Tibet | 2007 | KX421388 | IV |
| ^a^China/Tibet/Bharal/2008 | China/Tibet | 2008 | JX217850 | IV |
| Cote d'Ivoire 89 | Cote d'Ivoire | 1989 | EU267273 | I |
| Nigeria 76/1 | Nigeria | 1976 | EU267274 | II |
| Turkey 2000 | Turkey | 2000 | NC_006383 | IV |
| CIV 01 P 2009 | Cote d'Ivoire | 2009 | KR781451 | II |
| Ethiopia 1994 | Ethiopia | 1994 | KJ867540 | III |
| Ethiopia 2010 | Ethiopia | 2010 | KJ867541 | IV |
| Ghana NK1 2010 | Ghana | 2010 | KJ466104 | II |
| Oman 1983 | Oman | 1983 | KJ867544 | III |
| ^b^UAE 1986 | United Arab Emirates | 1986 | KJ867545 | III |
| India/TN/Gingee/2014 | India | 2014 | KR261605 | IV |
| Uganda 2012 | Uganda | 2012 | KJ867543 | III |
| ^c^Morocco 2008 | Morocco | 2008 | KC594074 | IV |
| China/XJYL/2013 | China | 2013 | KM091959 | IV |
| China/XJ2/2013 | China | 2013 | KX421384 | IV |
| China/XJ3/2013 | China | 2013 | KX421385 | IV |
| China/XJ4/2013 | China | 2013 | KX421386 | IV |
| China/XJ5/2013 | China | 2013 | KX421387 | IV |
| ^d^China/GS2014 | China | 2014 | MF443351 | IV |
| China/NX2014 | China | 2014 | MF443340 | IV |
| ChinaLN2014 | China | 2014 | MF443341 | IV |
| ChinaCQ2014 | China | 2014 | MF443353 | IV |
| ChinaHLJ2014 | China | 2014 | MF443346 | IV |
| ChinaYN2014 | China | 2014 | MF443336 | IV |
| ChinaSaX2014 | China | 2014 | MF443339 | IV |
| ChinaJX2014 | China | 2014 | MF443342 | IV |
| ChinaJL2014 | China | 2014 | MF443344 | IV |
| ChinaJS2014 | China | 2014 | MF443343 | IV |
| ChinaHeN2014 | China | 2014 | MF443347 | IV |
| ChinaHB2014 | China | 2014 | MF443348 | IV |
| ChinaAH2014 | China | 2014 | MF443354 | IV |
| ChinaSX2014 | China | 2014 | MF443337 | IV |
| ChinaGX2014 | China | 2014 | MF443350 | IV |
| ChinaGZ2014 | China | 2014 | MF443349 | IV |
| ChinaZJ2014 | China | 2014 | MF443335 | IV |
| ChinaHN2014 | China | 2014 | MF443345 | IV |
| ChinaGD2014 | China | 2014 | MF443352 | IV |
| ChinaSC2014 | China | 2014 | MF443338 | IV |
| PPRV-FY 2015 | China | 2015 | KX354359 | IV |
| PPRV/Mongolia/9/2016 | Mongolia | 2016 | KY888168 | IV |

a strain was isolated from wild bharal

b strain was isolated from dorcas gazelle

c strain was isolated from alpine goat

d strain was isolated from sheep

other strains were isolated from domestic goats

Table S2 Nucleotide usages in the six genes of PPRV

|  | F gene | H gene | L gene | M gene | N gene | P gene |
| --- | --- | --- | --- | --- | --- | --- |
| U% | 24.16±0.27 | 26.46±0.22 | 25.97±0.09 | 23.91±0.34 | 21.31±0.24 | 20.97±0.30 |
| C% | 21.93±0.22 | 22.22±0.17 | 21.97±0.11 | 22.34±0.23 | 23.21±0.21 | 23.78±0.24 |
| A% | 29.97±0.25 | 27.02±0.12 | 29.11±0.09 | 29.41±0.29 | 27.76±0.18 | 31.06±0.20 |
| G% | 23.95±0.20 | 24.30±0.17 | 22.95±0.09 | 24.35±0.22 | 27.73±0.17 | 24.19±0.28 |
| U1% | 17.28±0.31 | 19.08±0.24 | 20.84±0.19 | 17.76±0.32 | 15.67±0.32 | 15.18±0.25 |
| C1% | 18.02±0.23 | 20.17±0.15 | 19.11±0.16 | 18.37±0.43 | 18.93±0.28 | 19.22±0.31 |
| A1% | 33.96±0.28 | 30.98±0.29 | 31.81±0.14 | 32.18±0.17 | 29.00±0.32 | 30.35±0.25 |
| G1% | 30.74±0.24 | 29.76±0.26 | 28.24±0.08 | 31.69±0.33 | 36.40±0.27 | 35.25±0.23 |
| U2% | 31.82±0.30 | 32.35±0.25 | 30.67±0.04 | 32.72±0.15 | 28.69±0.13 | 22.68±0.19 |
| C2% | 23.97±0.69 | 21.24±0.29 | 20.45±0.05 | 18.82±0.20 | 23.60±0.11 | 24.53±0.23 |
| A2% | 27.15±0.29 | 27.38±0.18 | 31.15±0.06 | 29.39±0.24 | 28.56±0.12 | 36.02±0.37 |
| G2% | 17.07±0.32 | 19.03±0.16 | 17.74±0.10 | 19.07±0.20 | 19.14±0.12 | 16.76±0.29 |
| U3% | 23.38±0.56 | 27.96±0.59 | 26.40±0.31 | 21.23±0.95 | 19.57±0.76 | 25.06±0.85 |
| C3% | 23.80±0.83 | 25.26±0.44 | 26.35±0.38 | 29.83±0.99 | 27.08±0.69 | 27.58±0.63 |
| A3% | 28.79±0.76 | 22.69±0.46 | 24.36±0.29 | 26.64±0.89 | 25.70±0.56 | 26.81±0.31 |
| G3% | 24.03±0.34 | 24.10±0.62 | 22.89±0.31 | 22.30±0.66 | 27.64±0.37 | 20.55±0.47 |

Table S3 The average of CAI data in the six transcription units of PPRV

| Transcription unit | Average ± S.D. | Significance (p value) |
| --- | --- | --- |
| F | 0.599±0.010 | p = 1.33×10^-14^ |
| H | 0.641±0.005 |
| L | 0.635±0.003 |
| M | 0.647±0.006 |
| N | 0.661±0.007 |
| P | 0.644±0.005 |

Table S4 RSCU values for F gene of PPRV

|  | ^a^Group I | ^b^Group II | ^c^Group III | ^d^Group IV | ^e^Group V | ^f^Group VI |
| --- | --- | --- | --- | --- | --- | --- |
| **UUU(F)** | 1.05 | 0.74 | 1.01 | 0.97 | 1.12 | 1.00 |
| **UUC(F)** | 0.95 | 1.26 | 0.99 | 1.03 | 0.88 | 1.00 |
| **UUA(L)** | 0.83 | 0.65 | 0.78 | 0.88 | 0.89 | 0.98 |
| **UUG(L)** | 0.88 | 0.74 | 0.91 | **0.59** | **0.58** | **0.59** |
| **CUU(L)** | 1.13 | 1.20 | 0.94 | 1.27 | 1.26 | 1.28 |
| **CUC(L)** | 0.68 | 0.68 | 0.77 | 0.60 | 0.68 | **0.59** |
| **CUA(L)** | 1.13 | 1.10 | 1.12 | 0.94 | 0.87 | 0.79 |
| **CUG(L)** | 1.37 | **1.62** | 1.48 | **1.73** | **1.73** | **1.77** |
| **AUU(I)** | 0.73 | 0.72 | 0.69 | **0.58** | **0.54** | **0.54** |
| **AUC(I)** | 0.91 | 0.91 | 1.12 | 1.14 | 1.20 | 1.14 |
| **AUA(I)** | 1.37 | 1.38 | 1.19 | 1.28 | 1.26 | 1.32 |
| **GUU(V)** | 0.95 | 0.86 | 0.75 | 1.27 | 1.21 | 1.36 |
| **GUC(V)** | 1.08 | 1.12 | 1.26 | 0.73 | 0.84 | 0.64 |
| **GUA(V)** | 1.08 | 1.21 | 0.82 | 1.28 | 1.21 | 1.36 |
| **GUG(V)** | 0.90 | 0.81 | 1.18 | 0.73 | 0.74 | 0.65 |
| **UCU(S)** | **0.53** | 0.63 | 1.15 | **0.49** | **0.57** | **0.55** |
| **UCC(S)** | 0.99 | 0.73 | **0.40** | 1.01 | 0.86 | 0.95 |
| **UCA(S)** | **1.72** | **1.95** | **1.79** | **1.75** | **1.57** | **1.64** |
| **UCG(S)** | 0.66 | **0.55** | **0.52** | 0.61 | 0.86 | 0.68 |
| **AGU(S)** | 1.12 | 1.00 | 1.02 | 0.94 | 0.86 | 1.09 |
| **AGC(S)** | 0.99 | 1.14 | 1.12 | 1.22 | 1.29 | 1.09 |
| **CCU(P)** | 1.46 | 1.09 | 1.15 | 1.41 | 1.45 | 1.44 |
| **CCC(P)** | **0.37** | 0.73 | 0.93 | **0.40** | **0.36** | **0.37** |
| **CCA(P)** | **1.91** | **1.82** | **1.33** | **1.63** | **1.82** | **1.82** |
| **CCG(P)** | **0.27** | **0.36** | **0.59** | **0.56** | **0.36** | **0.36** |
| **ACU(T)** | 1.15 | 1.00 | 1.08 | 1.02 | 0.88 | 0.98 |
| **ACC(T)** | 0.97 | 1.16 | 1.25 | 1.04 | 1.17 | 1.07 |
| **ACA(T)** | 1.34 | 1.52 | 1.23 | **1.71** | **1.76** | **1.66** |
| **ACG(T)** | **0.56** | **0.32** | **0.44** | **0.23** | **0.20** | **0.29** |
| **GCU(A)** | 0.88 | 0.86 | 1.38 | 0.75 | 0.76 | 0.60 |
| **GCC(A)** | 1.28 | 1.12 | 1.13 | 1.11 | 1.05 | 1.20 |
| **GCA(A)** | 1.54 | **1.63** | 1.50 | **1.74** | **1.81** | **1.80** |
| **GCG(A)** | **0.31** | **0.38** | **0.00** | **0.40** | **0.38** | **0.39** |
| **UAU(Y)** | 0.85 | 0.93 | 0.78 | 0.79 | 0.80 | 0.81 |
| **UAC(Y)** | 1.15 | 1.07 | 1.22 | 1.21 | 1.20 | 1.19 |
| **CAU(H)** | 1.00 | 0.95 | 1.12 | 0.59 | **0.57** | **0.56** |
| **CAC(H)** | 1.00 | 1.05 | 0.88 | 1.41 | 1.43 | 1.44 |
| **CAA(Q)** | 0.69 | 0.73 | 0.94 | 0.64 | 0.71 | **0.59** |
| **CAG(Q)** | 1.31 | 1.27 | 1.06 | 1.36 | 1.29 | 1.41 |
| **AAU(N)** | 1.42 | 1.41 | 1.15 | 1.39 | 1.25 | 1.39 |
| **AAC(N)** | **0.59** | **0.59** | 0.85 | 0.61 | 0.75 | 0.61 |
| **AAA(K)** | 0.88 | 0.80 | 0.92 | 0.68 | 0.74 | 0.71 |
| **AAG(K)** | 1.13 | 1.20 | 1.08 | 1.32 | 1.26 | 1.29 |
| **GAU(D)** | 1.20 | 1.15 | 1.35 | 1.01 | 1.16 | 1.00 |
| **GAC(D)** | 0.80 | 0.85 | 0.65 | 0.99 | 0.84 | 1.00 |
| **GAA(E)** | 0.74 | 0.65 | 0.86 | 0.64 | **0.54** | 0.62 |
| **GAG(E)** | 1.26 | 1.35 | 1.14 | 1.36 | 1.46 | 1.38 |
| **UGU(C)** | 0.91 | 0.92 | 0.89 | 0.78 | 0.88 | 1.00 |
| **UGC(C)** | 1.10 | 1.09 | 1.12 | 1.23 | 1.13 | 1.00 |
| **CGU(R)** | **0.43** | **0.28** | **0.27** | **0.57** | 0.86 | **0.57** |
| **CGC(R)** | **0.56** | **0.56** | **0.54** | **0.33** | **0.00** | **0.29** |
| **CGA(R)** | **0.56** | **0.56** | 0.81 | 0.61 | **0.57** | **0.57** |
| **CGG(R)** | **0.26** | **0.56** | **0.20** | **0.52** | **0.57** | **0.57** |
| **AGA(R)** | **2.49** | **2.91** | **2.25** | **2.86** | **2.86** | **2.85** |
| **AGG(R)** | **1.70** | 1.12 | **1.95** | 1.12 | 1.14 | 1.16 |
| **GGU(G)** | **0.32** | **0.21** | **0.51** | **0.38** | **0.21** | **0.32** |
| **GGC(G)** | 0.80 | 0.89 | 0.69 | 0.77 | 0.92 | 0.84 |
| **GGA(G)** | 0.85 | 0.85 | 1.07 | 0.94 | 1.11 | 0.95 |
| **GGG(G)** | **2.03** | **2.05** | **1.73** | **1.91** | **1.77** | **1.89** |

**a** RSCU values were calculated for lineage I of PPRV (X74443, EU267273).

**b** RSCU values were calculated for lineage II of PPRV (EU267274, KR781451, KJ466104).

**c** RSCU values were calculated for lineage III of PPRV (KJ867540, KJ867544, KJ867545, KJ867543).

**d** RSCU values were calculated for lineage IV of PPRV except China (KJ867542, NC_006383, KJ867541, KR261605, KC594074, KY888168).

**e** RSCU values were calculated for lineage IV of PPRV in China during 2007-2008 (FJ905304, KX421388, JX217850, JF939201).

**f** RSCU values were calculated for lineage IV of PPRV in China during 2013-2015 (KM091959, KX421384, KX421385, KX421386, KX421387, MF443351, MF443340, MF443341, MF443353, MF443346, MF443336, MF443339, MF443342, MF443344, MF443343, MF443347, MF443348, MF443354, MF443337, MF443350, MF443349, MF443335, MF443345, MF443352, MF443338, KX354359).

Table S5 RSCU values for H gene of PPRV

|  | ^a^Group I | ^b^Group II | ^c^Group III | ^d^Group IV | ^e^Group V | ^f^Group VI |
| --- | --- | --- | --- | --- | --- | --- |
| **UUU(F)** | 1.04 | 0.90 | 0.80 | 0.87 | 0.92 | 0.92 |
| **UUC(F)** | 0.96 | 1.10 | 1.20 | 1.13 | 1.08 | 1.08 |
| **UUA(L)** | 0.69 | 0.71 | **0.53** | 0.64 | 0.62 | **0.54** |
| **UUG(L)** | 1.04 | 0.94 | 0.93 | 1.03 | 0.97 | 1.16 |
| **CUU(L)** | 1.00 | 0.94 | 0.95 | 1.12 | 1.06 | 1.16 |
| **CUC(L)** | 0.95 | 0.94 | 0.93 | 0.79 | 0.79 | 0.72 |
| **CUA(L)** | 0.70 | 0.85 | 1.04 | 0.82 | 0.79 | 0.89 |
| **CUG(L)** | **1.64** | **1.62** | **1.63** | **1.61** | **1.76** | 1.52 |
| **AUU(I)** | 0.91 | 1.01 | 1.30 | 1.02 | 0.93 | 0.91 |
| **AUC(I)** | 1.25 | 1.26 | 0.94 | 1.28 | 1.33 | 1.40 |
| **AUA(I)** | 0.85 | 0.73 | 0.76 | 0.71 | 0.73 | 0.70 |
| **GUU(V)** | 0.91 | 0.94 | 0.94 | 1.08 | 1.13 | 1.04 |
| **GUC(V)** | 1.07 | 1.08 | 0.85 | 1.01 | 1.00 | 1.04 |
| **GUA(V)** | 1.03 | 0.89 | 1.16 | 0.83 | 0.68 | 0.80 |
| **GUG(V)** | 0.99 | 1.09 | 1.07 | 1.08 | 1.19 | 1.12 |
| **UCU(S)** | **0.57** | 1.15 | 0.87 | 1.10 | 0.90 | 1.12 |
| **UCC(S)** | 1.35 | 1.10 | 1.08 | 1.10 | 1.25 | 1.11 |
| **UCA(S)** | **2.12** | **2.11** | **2.02** | **2.38** | **2.43** | **2.23** |
| **UCG(S)** | **0.07** | **0.00** | **0.33** | **0.00** | **0.00** | **0.00** |
| **AGU(S)** | 1.28 | 1.25 | 1.16 | 1.14 | 1.14 | 1.26 |
| **AGC(S)** | 0.64 | **0.39** | **0.54** | **0.29** | **0.29** | **0.28** |
| **CCU(P)** | **1.70** | 1.57 | **1.72** | 1.51 | 1.56 | 1.44 |
| **CCC(P)** | 0.62 | 0.75 | 0.93 | 0.94 | 0.89 | 1.00 |
| **CCA(P)** | 0.90 | 0.97 | 1.02 | 0.78 | 0.78 | 0.79 |
| **CCG(P)** | 0.79 | 0.71 | **0.32** | 0.78 | 0.78 | 0.78 |
| **ACU(T)** | 1.21 | 0.96 | 1.44 | 0.83 | 0.91 | 0.89 |
| **ACC(T)** | 1.35 | 1.37 | 1.26 | 1.43 | 1.37 | 1.40 |
| **ACA(T)** | 0.99 | 1.30 | 0.99 | 1.50 | 1.52 | 1.30 |
| **ACG(T)** | **0.45** | **0.37** | **0.31** | **0.24** | **0.21** | **0.40** |
| **GCU(A)** | 0.79 | 1.16 | 0.77 | 1.22 | 1.42 | 0.77 |
| **GCC(A)** | 1.57 | 1.51 | **1.83** | 1.41 | 1.33 | **1.71** |
| **GCA(A)** | 1.48 | 0.84 | 1.16 | 0.90 | 0.89 | 0.95 |
| **GCG(A)** | **0.16** | **0.48** | **0.25** | **0.47** | **0.36** | **0.57** |
| **UAU(Y)** | 0.73 | 0.73 | 1.00 | 0.96 | 0.95 | 0.95 |
| **UAC(Y)** | 1.28 | 1.27 | 1.00 | 1.04 | 1.05 | 1.05 |
| **CAU(H)** | 1.41 | 1.27 | 1.50 | 1.25 | 1.29 | 1.29 |
| **CAC(H)** | **0.60** | 0.73 | **0.51** | 0.76 | 0.71 | 0.71 |
| **CAA(Q)** | 0.82 | 0.88 | 0.81 | 0.82 | **0.50** | **0.50** |
| **CAG(Q)** | 1.18 | 1.12 | 1.19 | 1.18 | 1.50 | 1.50 |
| **AAU(N)** | 0.98 | 0.83 | 0.91 | 1.02 | 1.08 | 1.10 |
| **AAC(N)** | 1.03 | 1.17 | 1.10 | 0.98 | 0.92 | 0.90 |
| **AAA(K)** | 0.73 | 0.83 | 0.87 | 0.86 | 0.86 | 0.88 |
| **AAG(K)** | 1.28 | 1.17 | 1.14 | 1.14 | 1.14 | 1.12 |
| **GAU(D)** | 1.35 | 1.36 | 1.42 | 1.50 | 1.50 | 1.54 |
| **GAC(D)** | 0.66 | 0.64 | **0.59** | **0.50** | **0.50** | **0.46** |
| **GAA(E)** | 0.81 | 0.77 | 0.84 | 0.78 | 0.74 | 0.74 |
| **GAG(E)** | 1.19 | 1.23 | 1.17 | 1.22 | 1.27 | 1.26 |
| **UGU(C)** | 1.46 | 1.13 | 1.12 | 1.15 | 0.92 | 1.08 |
| **UGC(C)** | **0.54** | 0.87 | 0.88 | 0.85 | 1.08 | 0.92 |
| **CGU(R)** | **0.29** | **0.32** | **0.49** | **0.41** | **0.42** | **0.41** |
| **CGC(R)** | **0.36** | **0.41** | **0.28** | **0.39** | **0.42** | **0.41** |
| **CGA(R)** | **0.57** | **0.41** | **0.59** | **0.28** | **0.28** | **0.27** |
| **CGG(R)** | **0.51** | 0.64 | **0.56** | 0.71 | 0.70 | 0.82 |
| **AGA(R)** | **2.44** | **2.32** | **2.41** | **2.50** | **2.37** | **2.45** |
| **AGG(R)** | **1.85** | **1.91** | **1.67** | **1.73** | **1.81** | **1.64** |
| **GGU(G)** | 0.69 | 0.65 | **0.57** | **0.60** | **0.60** | **0.50** |
| **GGC(G)** | 1.08 | 1.10 | 1.15 | 1.18 | 1.20 | 1.20 |
| **GGA(G)** | 0.88 | 1.04 | 0.91 | 1.16 | 1.08 | 1.30 |
| **GGG(G)** | 1.37 | 1.21 | 1.38 | 1.08 | 1.13 | 1.00 |

**a** RSCU values were calculated for lineage I of PPRV (X74443, EU267273).

**b** RSCU values were calculated for lineage II of PPRV (EU267274, KR781451, KJ466104).

**c** RSCU values were calculated for lineage III of PPRV (KJ867540, KJ867544, KJ867545, KJ867543).

**d** RSCU values were calculated for lineage IV of PPRV except China (KJ867542, NC_006383, KJ867541, KR261605, KC594074, KY888168).

**e** RSCU values were calculated for lineage IV of PPRV in China during 2007-2008 (FJ905304, KX421388, JX217850, JF939201).

**f** RSCU values were calculated for lineage IV of PPRV in China during 2013-2015 (KM091959, KX421384, KX421385, KX421386, KX421387, MF443351, MF443340, MF443341, MF443353, MF443346, MF443336, MF443339, MF443342, MF443344, MF443343, MF443347, MF443348, MF443354, MF443337, MF443350, MF443349, MF443335, MF443345, MF443352, MF443338, KX354359).

Table S6 RSCU values for L gene of PPRV

|  | ^a^Group I | ^b^Group II | ^c^Group III | ^d^Group IV | ^e^Group V | ^f^Group VI |
| --- | --- | --- | --- | --- | --- | --- |
| **UUU(F)** | 0.89 | 0.96 | 0.84 | 0.92 | 0.93 | 0.94 |
| **UUC(F)** | 1.11 | 1.04 | 1.16 | 1.08 | 1.08 | 1.06 |
| **UUA(L)** | 0.82 | 0.72 | 0.91 | 0.74 | 0.70 | 0.78 |
| **UUG(L)** | 1.22 | 1.23 | 0.99 | 1.23 | 1.25 | 1.29 |
| **CUU(L)** | 0.95 | 1.07 | 1.16 | 1.10 | 1.14 | 1.14 |
| **CUC(L)** | 0.96 | 0.84 | 0.72 | 0.78 | 0.73 | 0.71 |
| **CUA(L)** | 0.89 | 0.92 | 0.88 | 1.02 | 1.03 | 0.99 |
| **CUG(L)** | 1.18 | 1.20 | 1.33 | 1.13 | 1.16 | 1.09 |
| **AUU(I)** | 0.82 | 0.74 | 0.82 | 0.69 | 0.71 | 0.65 |
| **AUC(I)** | 1.24 | 1.30 | 1.27 | 1.27 | 1.23 | 1.33 |
| **AUA(I)** | 0.95 | 0.96 | 0.91 | 1.04 | 1.06 | 1.02 |
| **GUU(V)** | 0.80 | 0.78 | 0.80 | 0.83 | 0.85 | 0.84 |
| **GUC(V)** | 1.28 | 1.20 | 1.36 | 1.26 | 1.22 | 1.19 |
| **GUA(V)** | 0.95 | 1.04 | 0.94 | 0.96 | 0.96 | 1.07 |
| **GUG(V)** | 0.98 | 0.99 | 0.90 | 0.95 | 0.97 | 0.90 |
| **UCU(S)** | 0.98 | 0.98 | 1.08 | 1.06 | 1.05 | 1.02 |
| **UCC(S)** | 1.21 | 1.15 | 1.20 | 1.15 | 1.18 | 1.18 |
| **UCA(S)** | 1.23 | 1.25 | 1.18 | 1.20 | 1.15 | 1.15 |
| **UCG(S)** | **0.51** | **0.51** | **0.38** | **0.51** | **0.49** | **0.59** |
| **AGU(S)** | 0.99 | 1.01 | 1.07 | 1.00 | 0.99 | 1.05 |
| **AGC(S)** | 1.09 | 1.11 | 1.09 | 1.08 | 1.14 | 1.02 |
| **CCU(P)** | 1.32 | 1.26 | 1.40 | 1.33 | 1.35 | 1.33 |
| **CCC(P)** | 1.02 | 0.91 | 0.77 | 0.80 | 0.76 | 0.78 |
| **CCA(P)** | 0.90 | 1.05 | 1.09 | 1.14 | 1.21 | 1.15 |
| **CCG(P)** | 0.78 | 0.78 | 0.75 | 0.74 | 0.67 | 0.74 |
| **ACU(T)** | 0.97 | 0.89 | 1.01 | 0.83 | 0.78 | 0.92 |
| **ACC(T)** | 1.27 | 1.36 | 1.31 | 1.40 | 1.40 | 1.30 |
| **ACA(T)** | 1.49 | 1.51 | 1.38 | 1.53 | 1.52 | 1.52 |
| **ACG(T)** | **0.27** | **0.24** | **0.30** | **0.24** | **0.32** | **0.25** |
| **GCU(A)** | 1.21 | 1.13 | 1.20 | 1.13 | 1.13 | 1.05 |
| **GCC(A)** | 1.31 | 1.36 | 1.30 | 1.32 | 1.35 | 1.37 |
| **GCA(A)** | 1.19 | 1.24 | 1.22 | 1.29 | 1.28 | 1.23 |
| **GCG(A)** | **0.30** | **0.26** | **0.28** | **0.27** | **0.25** | **0.35** |
| **UAU(Y)** | 0.95 | 0.96 | 1.02 | 1.07 | 1.07 | 1.05 |
| **UAC(Y)** | 1.06 | 1.04 | 0.98 | 0.93 | 0.93 | 0.95 |
| **CAU(H)** | 1.02 | 0.95 | 1.08 | 1.16 | 1.18 | 1.18 |
| **CAC(H)** | 0.98 | 1.05 | 0.92 | 0.85 | 0.82 | 0.82 |
| **CAA(Q)** | 0.93 | 0.97 | 1.04 | 0.98 | 1.02 | 1.05 |
| **CAG(Q)** | 1.07 | 1.03 | 0.97 | 1.02 | 0.98 | 0.95 |
| **AAU(N)** | 1.17 | 1.14 | 1.08 | 1.13 | 1.12 | 1.07 |
| **AAC(N)** | 0.83 | 0.86 | 0.92 | 0.87 | 0.88 | 0.93 |
| **AAA(K)** | 1.09 | 1.11 | 1.19 | 1.13 | 1.14 | 1.14 |
| **AAG(K)** | 0.92 | 0.89 | 0.81 | 0.87 | 0.86 | 0.86 |
| **GAU(D)** | 1.07 | 1.14 | 1.27 | 1.17 | 1.17 | 1.17 |
| **GAC(D)** | 0.93 | 0.86 | 0.73 | 0.83 | 0.83 | 0.83 |
| **GAA(E)** | 0.85 | 0.82 | 0.89 | 0.83 | 0.87 | 0.84 |
| **GAG(E)** | 1.16 | 1.18 | 1.12 | 1.17 | 1.13 | 1.16 |
| **UGU(C)** | 1.10 | 1.07 | 1.11 | 1.01 | 0.98 | 1.07 |
| **UGC(C)** | 0.90 | 0.93 | 0.89 | 0.99 | 1.02 | 0.93 |
| **CGU(R)** | **0.29** | **0.33** | **0.28** | **0.36** | **0.34** | **0.35** |
| **CGC(R)** | **0.34** | **0.35** | **0.45** | **0.34** | **0.34** | **0.35** |
| **CGA(R)** | 0.61 | 0.63 | **0.52** | 0.70 | 0.68 | 0.64 |
| **CGG(R)** | 0.66 | 0.74 | **0.54** | 0.73 | 0.73 | 0.79 |
| **AGA(R)** | **2.37** | **2.13** | **2.27** | **2.05** | **2.06** | **2.03** |
| **AGG(R)** | **1.74** | **1.83** | **1.93** | **1.82** | **1.85** | **1.83** |
| **GGU(G)** | 1.06 | 1.10 | 1.09 | 1.09 | 1.12 | 1.04 |
| **GGC(G)** | 0.80 | 0.81 | 0.87 | 0.78 | 0.76 | 0.84 |
| **GGA(G)** | 0.76 | 0.69 | 0.79 | 0.75 | 0.82 | 0.75 |
| **GGG(G)** | 1.39 | 1.40 | 1.26 | 1.39 | 1.30 | 1.37 |

**a** RSCU values were calculated for lineage I of PPRV (X74443, EU267273).

**b** RSCU values were calculated for lineage II of PPRV (EU267274, KR781451, KJ466104).

**c** RSCU values were calculated for lineage III of PPRV (KJ867540, KJ867544, KJ867545, KJ867543).

**d** RSCU values were calculated for lineage IV of PPRV except China (KJ867542, NC_006383, KJ867541, KR261605, KC594074, KY888168).

**e** RSCU values were calculated for lineage IV of PPRV in China during 2007-2008 (FJ905304, KX421388, JX217850, JF939201).

**f** RSCU values were calculated for lineage IV of PPRV in China during 2013-2015 (KM091959, KX421384, KX421385, KX421386, KX421387, MF443351, MF443340, MF443341, MF443353, MF443346, MF443336, MF443339, MF443342, MF443344, MF443343, MF443347, MF443348, MF443354, MF443337, MF443350, MF443349, MF443335, MF443345, MF443352, MF443338, KX354359).

Table S7 RSCU values for M gene of PPRV

|  | ^a^Group I | ^b^Group II | ^c^Group III | ^d^Group IV | ^e^Group V | ^f^Group VI |
| --- | --- | --- | --- | --- | --- | --- |
| **UUU(F)** | 0.93 | 0.80 | 0.93 | 0.94 | 1.07 | 1.04 |
| **UUC(F)** | 1.07 | 1.20 | 1.07 | 1.06 | 0.93 | 0.96 |
| **UUA(L)** | 0.77 | 0.76 | 1.22 | 1.12 | 0.88 | 1.09 |
| **UUG(L)** | 0.95 | 0.81 | 0.84 | 0.68 | 0.71 | 0.74 |
| **CUU(L)** | 0.77 | **0.46** | **0.50** | **0.43** | **0.35** | **0.38** |
| **CUC(L)** | **0.34** | 0.64 | 0.68 | 0.66 | 0.71 | 0.70 |
| **CUA(L)** | 1.63 | 1.63 | 1.01 | 1.30 | 1.41 | 1.32 |
| **CUG(L)** | 1.54 | **1.69** | **1.76** | **1.81** | **1.94** | **1.78** |
| **AUU(I)** | 0.89 | 0.82 | 0.97 | 0.85 | 0.75 | 0.81 |
| **AUC(I)** | 1.22 | 1.21 | 1.00 | 1.14 | 1.20 | 1.15 |
| **AUA(I)** | 0.89 | 0.97 | 1.04 | 1.02 | 1.05 | 1.04 |
| **GUU(V)** | **0.54** | 0.78 | 0.64 | 0.76 | 0.75 | 0.73 |
| **GUC(V)** | **1.90** | **1.65** | **1.78** | 1.56 | 1.50 | 1.55 |
| **GUA(V)** | 0.61 | 0.69 | **0.50** | 0.71 | 0.75 | 0.70 |
| **GUG(V)** | 0.95 | 0.87 | 1.08 | 0.98 | 1.00 | 1.02 |
| **UCU(S)** | 0.97 | 1.09 | 1.24 | 0.98 | 0.95 | 0.98 |
| **UCC(S)** | **0.34** | **0.10** | **0.26** | **0.33** | **0.32** | **0.33** |
| **UCA(S)** | **2.27** | **2.35** | **2.12** | **2.21** | **2.53** | **2.19** |
| **UCG(S)** | **0.00** | **0.00** | **0.26** | **0.17** | **0.00** | **0.33** |
| **AGU(S)** | 1.13 | 1.39 | 1.06 | 1.13 | 0.95 | 0.98 |
| **AGC(S)** | 1.30 | 1.07 | 1.06 | 1.18 | 1.26 | 1.21 |
| **CCU(P)** | 0.87 | 0.72 | 0.98 | 0.71 | 0.71 | 0.75 |
| **CCC(P)** | **1.95** | **1.86** | **2.09** | **2.16** | **2.35** | **2.32** |
| **CCA(P)** | 1.08 | 1.07 | 0.71 | 0.74 | **0.47** | **0.52** |
| **CCG(P)** | **0.11** | **0.35** | **0.22** | **0.40** | **0.47** | **0.41** |
| **ACU(T)** | 0.87 | 0.93 | 1.20 | 1.11 | 1.05 | 1.08 |
| **ACC(T)** | 1.18 | 1.28 | 1.40 | 1.42 | 1.47 | 1.45 |
| **ACA(T)** | 1.51 | 1.49 | 1.20 | 1.22 | 1.26 | 1.25 |
| **ACG(T)** | **0.44** | **0.28** | **0.20** | **0.24** | **0.21** | **0.21** |
| **GCU(A)** | 0.80 | 0.98 | 1.87 | 1.16 | 0.80 | 1.05 |
| **GCC(A)** | **1.60** | 1.42 | 1.07 | 1.42 | **1.60** | 1.48 |
| **GCA(A)** | **1.60** | 1.51 | 1.07 | 1.38 | **1.60** | 1.48 |
| **GCG(A)** | **0.00** | **0.09** | **0.00** | **0.05** | **0.00** | **0.00** |
| **UAU(Y)** | 0.85 | 0.72 | 0.92 | **0.54** | **0.31** | **0.45** |
| **UAC(Y)** | 1.16 | 1.28 | 1.08 | 1.46 | **1.69** | 1.55 |
| **CAU(H)** | 1.10 | 1.07 | 0.63 | 0.87 | 1.00 | 0.88 |
| **CAC(H)** | 0.90 | 0.93 | 1.38 | 1.13 | 1.00 | 1.12 |
| **CAA(Q)** | 0.66 | **0.58** | 0.75 | 0.75 | 0.75 | 0.75 |
| **CAG(Q)** | 1.34 | 1.42 | 1.25 | 1.25 | 1.25 | 1.25 |
| **AAU(N)** | 0.95 | 0.84 | 0.92 | 0.83 | 0.78 | 0.81 |
| **AAC(N)** | 1.06 | 1.16 | 1.08 | 1.18 | 1.22 | 1.19 |
| **AAA(K)** | 1.06 | 1.02 | 0.88 | 1.00 | 1.16 | 1.06 |
| **AAG(K)** | 0.94 | 0.98 | 1.13 | 1.01 | 0.84 | 0.95 |
| **GAU(D)** | 1.48 | 1.51 | 1.20 | 1.37 | 1.37 | 1.41 |
| **GAC(D)** | **0.53** | **0.49** | 0.80 | **0.63** | **0.63** | **0.59** |
| **GAA(E)** | 0.80 | 0.85 | **0.47** | 0.75 | 0.81 | 0.71 |
| **GAG(E)** | 1.20 | 1.15 | 1.53 | 1.25 | 1.19 | 1.29 |
| **UGU(C)** | **0.00** | **0.13** | **0.40** | **0.40** | **0.40** | **0.40** |
| **UGC(C)** | **2.00** | **1.87** | **1.60** | **1.60** | **1.60** | **1.60** |
| **CGU(R)** | **0.24** | **0.24** | **0.23** | **0.24** | **0.25** | **0.24** |
| **CGC(R)** | **0.59** | 0.71 | **0.47** | 0.64 | 0.75 | 0.66 |
| **CGA(R)** | 0.94 | 0.87 | 0.70 | 0.72 | 0.75 | 0.71 |
| **CGG(R)** | **0.24** | **0.24** | **0.23** | **0.24** | **0.25** | **0.24** |
| **AGA(R)** | **2.71** | **2.69** | **2.80** | **2.78** | **2.50** | **2.67** |
| **AGG(R)** | 1.29 | 1.26 | 1.58 | 1.39 | 1.50 | 1.48 |
| **GGU(G)** | 0.87 | 1.04 | 0.73 | 0.89 | 0.87 | 0.83 |
| **GGC(G)** | 0.87 | 0.81 | 0.65 | 0.87 | 1.04 | 1.10 |
| **GGA(G)** | 1.40 | 1.28 | 1.42 | 1.38 | 1.39 | 1.26 |
| **GGG(G)** | 0.87 | 0.87 | 1.21 | 0.87 | 0.70 | 0.82 |

**a** RSCU values were calculated for lineage I of PPRV (X74443, EU267273).

**b** RSCU values were calculated for lineage II of PPRV (EU267274, KR781451, KJ466104).

**c** RSCU values were calculated for lineage III of PPRV (KJ867540, KJ867544, KJ867545, KJ867543).

**d** RSCU values were calculated for lineage IV of PPRV except China (KJ867542, NC_006383, KJ867541, KR261605, KC594074, KY888168).

**e** RSCU values were calculated for lineage IV of PPRV in China during 2007-2008 (FJ905304, KX421388, JX217850, JF939201).

**f** RSCU values were calculated for lineage IV of PPRV in China during 2013-2015 (KM091959, KX421384, KX421385, KX421386, KX421387, MF443351, MF443340, MF443341, MF443353, MF443346, MF443336, MF443339, MF443342, MF443344, MF443343, MF443347, MF443348, MF443354, MF443337, MF443350, MF443349, MF443335, MF443345, MF443352, MF443338, KX354359).

Table S8 RSCU values for N gene of PPRV

|  | ^a^Group I | ^b^Group II | ^c^Group III | ^d^Group IV | ^e^Group V | ^f^Group VI |
| --- | --- | --- | --- | --- | --- | --- |
| **UUU(F)** | 0.93 | 1.18 | 1.15 | 1.16 | 1.18 | 1.18 |
| **UUC(F)** | 1.08 | 0.82 | 0.86 | 0.84 | 0.82 | 0.82 |
| **UUA(L)** | **0.57** | **0.35** | **0.41** | **0.44** | **0.35** | **0.57** |
| **UUG(L)** | 0.90 | 1.27 | 1.27 | 1.27 | 1.27 | 1.25 |
| **CUU(L)** | 0.54 | 0.81 | 0.81 | 0.83 | 0.81 | 0.91 |
| **CUC(L)** | **1.78** | **1.73** | **1.68** | **1.67** | **1.73** | 1.58 |
| **CUA(L)** | **0.48** | 0.69 | 0.69 | 0.65 | 0.69 | **0.57** |
| **CUG(L)** | **1.75** | 1.15 | 1.15 | 1.13 | 1.15 | 1.13 |
| **AUU(I)** | **0.49** | 0.97 | 0.92 | 0.95 | 0.97 | 0.95 |
| **AUC(I)** | 1.47 | 1.30 | 1.34 | 1.36 | 1.30 | 1.50 |
| **AUA(I)** | 1.05 | 0.73 | 0.75 | 0.68 | 0.73 | **0.56** |
| **GUU(V)** | 0.94 | 1.20 | 1.15 | 1.14 | 1.23 | 1.14 |
| **GUC(V)** | 0.80 | **1.60** | **1.70** | **1.71** | **1.65** | **1.69** |
| **GUA(V)** | 0.93 | **0.53** | **0.54** | **0.55** | **0.55** | **0.59** |
| **GUG(V)** | 1.34 | 0.67 | 0.61 | **0.59** | **0.58** | **0.57** |
| **UCU(S)** | **0.46** | **0.57** | 0.64 | 0.67 | **0.57** | **0.58** |
| **UCC(S)** | 0.72 | 1.43 | 1.36 | 1.34 | 1.43 | 1.42 |
| **UCA(S)** | **2.02** | **1.57** | **1.64** | **1.66** | **1.57** | **1.71** |
| **UCG(S)** | **1.81** | 1.14 | 1.07 | 1.00 | 1.14 | 0.87 |
| **AGU(S)** | **0.53** | 0.71 | 0.71 | 0.76 | 0.71 | 0.85 |
| **AGC(S)** | **0.46** | **0.57** | **0.57** | **0.57** | **0.57** | **0.57** |
| **CCU(P)** | 0.98 | 0.87 | 0.87 | 0.87 | 0.87 | 0.91 |
| **CCC(P)** | 1.06 | 1.39 | 1.39 | 1.32 | 1.39 | 1.10 |
| **CCA(P)** | 1.16 | 1.22 | 1.22 | 1.24 | 1.22 | 1.44 |
| **CCG(P)** | 0.81 | **0.52** | **0.52** | **0.57** | **0.52** | **0.55** |
| **ACU(T)** | 1.11 | 1.22 | 1.28 | 1.24 | 1.22 | 1.22 |
| **ACC(T)** | 1.20 | 1.39 | 1.36 | 1.38 | 1.39 | 1.39 |
| **ACA(T)** | 1.19 | 0.87 | 0.85 | 0.89 | 0.87 | 1.03 |
| **ACG(T)** | **0.51** | **0.52** | **0.51** | **0.49** | **0.52** | **0.36** |
| **GCU(A)** | 0.89 | 0.89 | 0.93 | 0.88 | 0.88 | 0.85 |
| **GCC(A)** | 1.38 | 0.98 | 0.97 | 1.02 | 0.97 | 1.02 |
| **GCA(A)** | 1.25 | **1.60** | 1.59 | 1.56 | 1.58 | 1.53 |
| **GCG(A)** | **0.49** | **0.53** | **0.53** | **0.55** | **0.59** | **0.60** |
| **UAU(Y)** | 1.39 | 1.45 | 1.45 | 1.45 | 1.49 | 1.45 |
| **UAC(Y)** | 0.61 | **0.55** | **0.55** | **0.55** | **0.51** | **0.55** |
| **CAU(H)** | **0.34** | **0.00** | **0.00** | **0.00** | **0.00** | **0.00** |
| **CAC(H)** | **1.67** | **2.00** | **2.00** | **2.00** | **2.00** | **2.00** |
| **CAA(Q)** | 0.86 | 0.92 | 0.92 | 0.90 | 0.92 | 0.92 |
| **CAG(Q)** | 1.14 | 1.08 | 1.08 | 1.10 | 1.08 | 1.08 |
| **AAU(N)** | 1.15 | **0.30** | **0.31** | **0.31** | **0.30** | **0.32** |
| **AAC(N)** | 0.85 | **1.70** | **1.69** | **1.69** | **1.70** | **1.68** |
| **AAA(K)** | **0.58** | 0.95 | 0.95 | 0.93 | 0.95 | 0.86 |
| **AAG(K)** | 1.43 | 1.05 | 1.05 | 1.07 | 1.05 | 1.14 |
| **GAU(D)** | 1.10 | 0.76 | 0.74 | 0.72 | 0.78 | 0.67 |
| **GAC(D)** | 0.91 | 1.24 | 1.27 | 1.28 | 1.22 | 1.33 |
| **GAA(E)** | **0.42** | 0.84 | 0.84 | 0.81 | 0.84 | 0.76 |
| **GAG(E)** | 1.58 | 1.16 | 1.17 | 1.20 | 1.16 | 1.24 |
| **UGU(C)** | 1.00 | **0.00** | **0.00** | **0.00** | **0.00** | **0.00** |
| **UGC(C)** | 1.00 | **2.00** | **2.00** | **2.00** | **2.00** | **2.00** |
| **CGU(R)** | **0.53** | **0.60** | **0.60** | **0.60** | **0.60** | **0.60** |
| **CGC(R)** | **0.30** | **0.60** | **0.60** | **0.60** | **0.60** | **0.60** |
| **CGA(R)** | **0.53** | **0.60** | **0.53** | **0.50** | **0.60** | **0.45** |
| **CGG(R)** | **0.38** | **0.30** | **0.38** | **0.40** | **0.30** | **0.45** |
| **AGA(R)** | **1.90** | **1.95** | **2.03** | **2.03** | **1.95** | **2.10** |
| **AGG(R)** | **2.36** | **1.95** | **1.88** | **1.88** | **1.95** | **1.80** |
| **GGU(G)** | **0.60** | **0.53** | **0.53** | **0.51** | **0.53** | **0.57** |
| **GGC(G)** | 0.98 | 0.62 | 0.62 | 0.62 | 0.62 | **0.53** |
| **GGA(G)** | 1.05 | 1.42 | 1.45 | 1.46 | 1.42 | 1.51 |
| **GGG(G)** | 1.38 | 1.42 | 1.41 | 1.40 | 1.42 | 1.38 |

**a** RSCU values were calculated for lineage I of PPRV (X74443, EU267273).

**b** RSCU values were calculated for lineage II of PPRV (EU267274, KR781451, KJ466104).

**c** RSCU values were calculated for lineage III of PPRV (KJ867540, KJ867544, KJ867545, KJ867543).

**d** RSCU values were calculated for lineage IV of PPRV except China (KJ867542, NC_006383, KJ867541, KR261605, KC594074, KY888168).

**e** RSCU values were calculated for lineage IV of PPRV in China during 2007-2008 (FJ905304, KX421388, JX217850, JF939201).

**f** RSCU values were calculated for lineage IV of PPRV in China during 2013-2015 (KM091959, KX421384, KX421385, KX421386, KX421387, MF443351, MF443340, MF443341, MF443353, MF443346, MF443336, MF443339, MF443342, MF443344, MF443343, MF443347, MF443348, MF443354, MF443337, MF443350, MF443349, MF443335, MF443345, MF443352, MF443338, KX354359).

Table S9 RSCU values for P gene of PPRV

|  | ^a^Group I | ^b^Group II | ^c^Group III | ^d^Group IV | ^e^Group V | ^f^Group VI |
| --- | --- | --- | --- | --- | --- | --- |
| **UUU(F)** | 1.00 | 0.92 | 1.04 | 1.21 | 1.14 | 1.42 |
| **UUC(F)** | 1.00 | 1.08 | 0.97 | 0.79 | 0.86 | **0.58** |
| **UUA(L)** | 0.61 | 0.75 | 0.92 | 0.66 | 0.77 | **0.46** |
| **UUG(L)** | 0.76 | **0.55** | **0.57** | **0.53** | **0.46** | 0.62 |
| **CUU(L)** | 1.44 | 1.15 | 1.42 | 1.04 | 0.92 | 1.09 |
| **CUC(L)** | 1.53 | **1.65** | **1.87** | **1.86** | **2.15** | **1.85** |
| **CUA(L)** | 1.06 | 1.30 | 0.84 | 1.40 | 1.08 | 1.53 |
| **CUG(L)** | **0.60** | **0.60** | **0.38** | **0.51** | 0.62 | **0.46** |
| **AUU(I)** | 0.74 | 0.70 | 0.72 | 0.73 | 0.71 | 0.69 |
| **AUC(I)** | 1.38 | 1.50 | 1.34 | 1.38 | 1.34 | 1.38 |
| **AUA(I)** | 0.89 | 0.81 | 0.95 | 0.88 | 0.95 | 0.92 |
| **GUU(V)** | 1.24 | 1.08 | 1.26 | 0.77 | 0.86 | 0.71 |
| **GUC(V)** | **1.69** | **1.85** | **1.70** | **2.06** | **2.00** | **2.14** |
| **GUA(V)** | **0.16** | **0.15** | **0.07** | **0.17** | **0.14** | **0.14** |
| **GUG(V)** | 0.93 | 0.93 | 0.98 | 1.01 | 1.00 | 1.00 |
| **UCU(S)** | **1.62** | **1.67** | **1.85** | 1.45 | 1.41 | 1.43 |
| **UCC(S)** | 1.18 | 1.24 | 0.97 | 1.47 | 1.50 | 1.43 |
| **UCA(S)** | 1.35 | 1.24 | 1.34 | 1.25 | 1.13 | 1.33 |
| **UCG(S)** | **0.23** | **0.25** | **0.23** | **0.30** | **0.38** | **0.38** |
| **AGU(S)** | 0.73 | 0.65 | 0.89 | **0.51** | **0.47** | **0.57** |
| **AGC(S)** | 0.91 | 0.96 | 0.74 | 1.03 | 1.13 | 0.86 |
| **CCU(P)** | 1.14 | 1.01 | 1.00 | 0.94 | 0.92 | 1.12 |
| **CCC(P)** | 0.91 | 0.93 | 0.97 | 0.82 | 0.62 | 0.81 |
| **CCA(P)** | 1.14 | 1.23 | 1.10 | 1.40 | 1.69 | 1.28 |
| **CCG(P)** | 0.83 | 0.82 | 0.94 | 0.84 | 0.77 | 0.80 |
| **ACU(T)** | 0.65 | 1.11 | 0.74 | 0.99 | 0.76 | 1.05 |
| **ACC(T)** | 1.43 | 1.10 | 1.46 | 1.18 | 1.33 | 1.21 |
| **ACA(T)** | 1.37 | 1.29 | 1.40 | 1.35 | 1.52 | 1.22 |
| **ACG(T)** | **0.55** | **0.49** | **0.40** | **0.48** | **0.38** | **0.52** |
| **GCU(A)** | 0.93 | 1.00 | 1.08 | 1.09 | 1.16 | 0.96 |
| **GCC(A)** | 0.72 | 1.00 | 1.12 | 0.91 | 0.90 | 0.98 |
| **GCA(A)** | 1.86 | 1.59 | 1.51 | 1.57 | 1.55 | 1.66 |
| **GCG(A)** | **0.50** | **0.41** | **0.29** | **0.44** | **0.39** | **0.41** |
| **UAU(Y)** | 1.22 | 1.44 | 1.34 | 1.35 | 1.40 | 1.25 |
| **UAC(Y)** | 0.79 | **0.56** | 0.66 | 0.66 | 0.60 | 0.75 |
| **CAU(H)** | 1.26 | 1.19 | 1.14 | 1.03 | 1.00 | 1.09 |
| **CAC(H)** | 0.74 | 0.81 | 0.86 | 0.97 | 1.00 | 0.91 |
| **CAA(Q)** | 0.98 | 0.96 | 1.14 | 0.97 | 0.96 | 0.87 |
| **CAG(Q)** | 1.02 | 1.04 | 0.87 | 1.03 | 1.04 | 1.13 |
| **AAU(N)** | 0.95 | 0.83 | 0.80 | 0.84 | 0.82 | 1.00 |
| **AAC(N)** | 1.06 | 1.17 | 1.20 | 1.16 | 1.18 | 1.00 |
| **AAA(K)** | 1.01 | 0.95 | 0.89 | 0.84 | 0.76 | 0.82 |
| **AAG(K)** | 0.99 | 1.05 | 1.11 | 1.17 | 1.24 | 1.18 |
| **GAU(D)** | 1.13 | 1.17 | 1.00 | 1.20 | 1.23 | 1.16 |
| **GAC(D)** | 0.87 | 0.83 | 1.00 | 0.80 | 0.78 | 0.84 |
| **GAA(E)** | 0.73 | 0.78 | 0.83 | 0.82 | 0.78 | 0.89 |
| **GAG(E)** | 1.27 | 1.22 | 1.17 | 1.18 | 1.22 | 1.11 |
| **UGU(C)** | 1.50 | 1.50 | 1.25 | 1.45 | 1.33 | 1.50 |
| **UGC(C)** | **0.50** | **0.50** | 0.75 | **0.55** | 0.67 | **0.50** |
| **CGU(R)** | 1.05 | 1.04 | 1.13 | 1.20 | 1.19 | 1.13 |
| **CGC(R)** | **0.30** | **0.39** | **0.25** | **0.30** | **0.30** | **0.29** |
| **CGA(R)** | **0.45** | **0.57** | **0.44** | **0.30** | **0.30** | **0.29** |
| **CGG(R)** | **0.45** | **0.29** | **0.32** | **0.35** | **0.30** | **0.29** |
| **AGA(R)** | **2.40** | **2.57** | **2.99** | **3.10** | **3.33** | **3.15** |
| **AGG(R)** | 1.35 | 1.14 | 0.88 | 0.75 | **0.59** | 0.86 |
| **GGU(G)** | 0.73 | **0.60** | 0.90 | **0.47** | **0.50** | **0.55** |
| **GGC(G)** | 0.87 | 0.95 | 0.77 | 1.06 | 0.95 | 1.09 |
| **GGA(G)** | 1.59 | 1.48 | 1.59 | 1.48 | **1.61** | 1.27 |
| **GGG(G)** | 0.82 | 0.97 | 0.74 | 0.99 | 0.95 | 1.09 |

**a** RSCU values were calculated for lineage I of PPRV (X74443, EU267273).

**b** RSCU values were calculated for lineage II of PPRV (EU267274, KR781451, KJ466104).

**c** RSCU values were calculated for lineage III of PPRV (KJ867540, KJ867544, KJ867545, KJ867543).

**d** RSCU values were calculated for lineage IV of PPRV except China (KJ867542, NC_006383, KJ867541, KR261605, KC594074, KY888168).

**e** RSCU values were calculated for lineage IV of PPRV in China during 2007-2008 (FJ905304, KX421388, JX217850, JF939201).

**f** RSCU values were calculated for lineage IV of PPRV in China during 2013-2015 (KM091959, KX421384, KX421385, KX421386, KX421387, MF443351, MF443340, MF443341, MF443353, MF443346, MF443336, MF443339, MF443342, MF443344, MF443343, MF443347, MF443348, MF443354, MF443337, MF443350, MF443349, MF443335, MF443345, MF443352, MF443338, KX354359).
